# Silicon-mediated drought tolerance in *Vigna mungo* through coordinated regulation of aquaporin genes, plant water relations and photosynthetic processes

**DOI:** 10.1038/s41598-026-59877-x

**Published:** 2026-07-09

**Authors:** Zeba Ali, Syed Riaz Ahmed, Iram Ijaz, Abdul Haleem, Muhammad Uzair Siddique, Jahangir Khan, Milan Skalicky, Yongming Liu, Ijaz Rasul, Daniel K. Y. Tan

**Affiliations:** 1 Department of Plant Breeding and Genetics, University of Agriculture Faisalabad, Faisalabad, 38000 Pakistan; 2 Pakistan Agricultural Research Council, Balochistan Agricultural Research and Development Centre (BARDC), Quetta, Pakistan; 3 Faculty of Agriculture, University of Kunduz, Kunduz, Afghanistan; 4 University of Florida, Gainesville, USA; 5 Directorate of Agriculture Research, Killa Saifullah, Balochistan, Pakistan; 6 Department of Botany and Plant Physiology, Faculty of Agrobiology, Food and Natural Resources, Czech University of Life Sciences Prauge, 165 00 Prague, Czech Republic; 7 National Nanfan Research Institute (Sanya), Chinese Academy of Agricultural Sciences, Sanya, China; 8 Department of Bioinformatics and Biotechnology, Government College University Faisalabad, Faisalabad, 38000 Pakistan; 9 Faculty of Science, Plant Breeding Institute, School of Life and Environmental Sciences, Sydney Institute of Agriculture, The University of Sydney, Sydney, NSW 2006 Australia

**Keywords:** Mash bean, Drought stress, Silicon, Gas exchange, Osmotic potential, Aquaporin genes, Physiology, Plant sciences

## Abstract

Drought stress restricts growth and productivity in grain legumes, yet the integrative mechanisms underlying silicon-mediated drought tolerance in *Vigna mungo* (mash bean) remain poorly understood. The present study aimed to investigate the role of silicon in mitigating drought-induced damage in two mash bean cultivars. To evaluate this effect, plants were subjected to different drought regimes with and without silicon. Data on growth traits, biomass accumulation, reproductive attributes, photosynthetic gas exchange parameters, water relations, antioxidant enzyme activities, phenolic compounds, nutrient dynamics, and expression of drought-responsive genes were collected and statistically analyzed. Multivariate analyses, including PCA and structural equation modeling (SEM), were used to elucidate relationships among measured variables. Drought significantly reduced plant growth, biomass production, and reproductive traits, accompanied by marked declines in net assimilation rate (up to 35–40%), stomatal conductance, and transpiration under 25% field capacity (FC). In contrast, drought increased WUE (49.7–56.2 µmol CO₂ mmol⁻^1^ H₂O), antioxidant enzyme activities, and phenolic accumulation. Silicon supplementation substantially improved plant growth, restored photosynthetic performance, and enhanced antioxidant defense under drought conditions. Si-treated plants exhibited increased activities of SOD, CAT, and APX, higher phenolic content, improved water status, and upregulated expression of *DREB2A*, *PIP2-1*, and *TIP4-1* compared with non-supplemented plants. Multivariate analyses clearly separated drought and Si treatments, while SEM revealed strong relationships linking gene expression, biochemical defense systems, physiological processes, and plant growth responses. In conclusion, silicon supplementation significantly enhances drought tolerance in mash bean by improving photosynthetic efficiency, antioxidant capacity, and molecular regulation of water transport.

**Supplementary Information:**

The online version contains supplementary material available at https://doi.org/10.1038/s41598-026-59877-x.

## Introduction

The current climate change is altering cropping pattern, crop cycle, and growth and development by increasing the frequency and duration of drought stress due to reduced rainfall, particularly in semi-arid and arid regions worldwide^1^. Balochista, the largest province of Pakistan by area, has been strongly affected by the current climate change, which is inducing severe and prolonged heat, drought and other abiotic stresses^2^. The underground freshwater resources have declined to historically low levels, raising concerns about sustainable agriculture and regional food security. Drought stress is one of the most severe environmental constraints affecting agricultural productivity worldwide^3,4^. Drought stress is known to affect plant growth and yield by disrupting physiological processes, impairing photosynthesis, altering nutrient balance and uptake, and inducing oxidative damage through excessive generation of reactive oxygen species (ROS)^5^. At the cellular level, drought stress reduces turgor pressure, restricts cell expansion, and interferes with metabolic homeostasis, ultimately leading to reduced biomass accumulation and yield losses. Consequently, improving drought tolerance in crops has become a major priority for sustainable agriculture and global food security^6^.

Among grain legumes, *Vigna mungo* (L.) Hepper, commonly known as mash bean or black gram, is an important pulse crop worldwide cultivated across South and Southeast Asia. The crop plays a critical role in human nutrition due to its high protein content and its contribution of essential amino acids, vitamins, and minerals. In addition, mash bean contributes to soil fertility through biological nitrogen fixation, making it an important component of sustainable cropping systems^7^. Despite its agronomic and nutritional importance, mash bean productivity remains relatively low, largely due to its sensitivity to abiotic stresses, particularly drought. Water scarcity during vegetative and reproductive stages can severely reduce plant growth, photosynthetic activity, and reproductive development, resulting in significant yield reductions^8^. Therefore, understanding the physiological and molecular mechanisms underlying drought tolerance in mash bean is essential for developing effective management strategies and improving crop resilience. Plants employ a complex network of adaptive responses to cope with drought stress^9,10^. These responses include morphological adjustments, such as reduced leaf area and altered biomass allocation; physiological changes such as stomatal regulation and improved water use efficiency, and biochemical mechanisms, including the activation of antioxidant defense systems. Drought stress typically leads to the ROS accumulation, which can cause oxidative damage to cellular components including membranes, proteins, and nucleic acids^11^. To mitigate oxidative stress, plants activate both enzymatic antioxidants, such as superoxide dismutase, catalase, and ascorbate peroxidase, and non-enzymatic compounds including phenolics and flavonoids. Additionally, osmolytes such as proline and soluble proteins contribute to osmotic adjustment and cellular stabilization under water-deficit conditions^12^. At the molecular level, drought-responsive transcription factors and membrane transport proteins, including aquaporins, play crucial roles in regulating water transport and stress signaling pathways^12^.

In recent years, silicon (Si) has gained increasing attention as a beneficial element that enhances plant tolerance to various abiotic stresses. Although not considered an essential nutrient for most plants, Si can improve plant resistance to drought, salinity, and temperature stress^13^. Si-mediated drought tolerance has been attributed to multiple mechanisms, including improved water uptake, enhanced antioxidant defense, stabilization of photosynthetic machinery, and reinforcement of cell walls^14^. Moreover, Si may influence the expression of drought-responsive genes, including transcription factors and aquaporins that regulate water transport and stress signaling pathways. For instance, dehydration-responsive element-binding (*DREB*) transcription factors are known to regulate downstream stress-protective genes. At the same time, aquaporin proteins such as plasma membrane intrinsic proteins (*PIPs*) and tonoplast intrinsic proteins (*TIPs*) facilitate water movement across cellular membranes, thereby maintaining cellular water balance under stress conditions^15,16^. Despite the documented benefits of Si for legume drought resilience, the fundamental mechanisms linking Si-regulated plant hydraulics to molecular water transport pathways in mash bean have yet to be fully elucidated. Previous studies in this crop have largely focused on growth performance, antioxidant responses, or nutrient dynamics under drought conditions^17–20^. However, the integration of plant hydraulic regulation, photosynthetic performance, and aquaporin-mediated water transport under Si supplementation has not been comprehensively investigated. Aquaporins, particularly *PIPs* and *TIPs*, play crucial roles in regulating membrane water permeability and maintaining cellular water balance during water deficit. Understanding how Si influences these molecular water channels and their downstream effects on plant water relations and photosynthetic processes is therefore essential for elucidating the physiological mechanisms underlying silicon-mediated drought tolerance. Moreover, a comparative evaluation of mash bean cultivars with contrasting drought responses under varying levels of water deficit and Si supplementation remains lacking. Although silicon-mediated drought tolerance has been reported in several crop species, the molecular mechanisms linking aquaporin regulation, plant water relations, and photosynthetic performance remain poorly understood in *Vigna mungo*, particularly under varying drought intensities. To the best of our knowledge, no previous study has examined the coordinated regulation of aquaporin gene expression, plant water relations, and photosynthetic gas exchange in mash bean under drought stress in the context of Si supplementation. Addressing this knowledge gap is particularly important for understanding how Si improves hydraulic functioning and physiological stability in drought-prone environments. Moreover, Structural Equation Modeling (SEM) has emerged as a powerful statistical tool for analyzing complex causal relationships among biological variables^21^. SEM allows researchers to simultaneously evaluate direct and indirect interactions among molecular, biochemical, physiological, and agronomic traits within a unified framework. In plant stress physiology, SEM is particularly valuable because it enables the identification of hierarchical regulatory pathways that link gene expression to downstream metabolic responses, physiological performance, and ultimately crop productivity. By integrating multiple datasets, SEM provides deeper insights into the mechanisms by which plants coordinate stress-adaptation processes and maintain productivity under adverse environmental conditions^22,23^.

Therefore, the present study aimed to investigate the role of Si in enhancing drought tolerance in two cultivars of *Vigna mungo* through an integrated physiological and molecular approach. Specifically, the study examined the effects of different drought stress levels and Si supplementation on plant growth, yield-related traits, water relations, and photosynthetic performance. In addition, the expression of key aquaporin genes (*PIP2-1* and *TIP4-1*) and the drought-responsive transcription factor *DREB2A* was evaluated to better understand their involvement in plant hydraulic and physiological responses under water deficit conditions. Furthermore, multivariate analyses and structural equation modeling were employed to integrate molecular, biochemical, and physiological responses and elucidate the regulatory mechanisms underlying Si-mediated drought tolerance. This integrative approach provides new insights into the role of aquaporin-mediated water transport, plant water relations, and photosynthetic regulation in improving drought tolerance in mash bean.

## Material and methods

### Experimental site, design and growing conditions

The current experiment was conducted at PARC-BARDC-Horticulture Research Institute (HRI), Balochistan, Pakistan. Seeds of two *Vigna mungo* cultivars, “Chakwal Mash” and “Arooj” were obtained from the Balochistan Agricultural Research and Development Centre, Quetta. Two commercially and widely cultivated *Vigna mungo* L. cultivars, Chakwal Mash and Arooj, were selected for this study due to their broad agricultural relevance in Pakistan and their contrasting adaptation characteristics. Chakwal Mash, developed by the Barani Agricultural Research Institute (BARI), Chakwal, is well adapted to rain-fed and drought-prone environments, whereas Arooj Mash (Mash Urooj-2011), developed by the Pulses Research Institute, Faisalabad, is recognized for its comparatively shorter crop duration and widespread cultivation under diverse production systems. Selecting these cultivars enables the evaluation of Si-mediated drought responses across genotypes with differing adaptations to water-limited environments. Seeds were surfaced-sterilize with 10% (v/v) sodium hypochlorite for 10 min, rinsed thoroughly with distilled water, and sown in plastic pots (6 cm in diameter × 13 cm in depth) filled with homogenized soil. Five seeds were sown per pot and thinned to one uniform plant per pot after emergence. Plants were grown in a green-net house under natural photoperiod. The experiment was laid out in a factorial completely randomized design with three replications and seven treatments. The treatments included, T1: 100% Field Capacity (FC), T2: 75% FC, T3: 50% FC, T4: 25% FC, T5: 25% FC + 1 mM silicon (Si), T6: 50% FC + 1 mM Si and T7: 75% FC + 1 mM Si. The applied Si concentration (1 mM) was selected based on optimization screening from preliminary pilot trials in our laboratory and supported by extensive literature on leguminous and other crops. In legumes, 1 mM Si represents a physiological threshold that effectively stimulates root hydraulic conductivity, osmolyte accumulation, and antioxidant enzyme activity without triggering osmotic imbalances or phytotoxic reactions that typically occur at higher alkaline concentrations (e.g., > 2 mM). Si supplementation was applied only under drought treatments to specifically investigate its role in alleviating drought-induced disruptions in plant water relations, hydraulic regulation, and photosynthetic performance. Si was supplied as orthosilicic acid (H_4_SiO_4_; Sigma-Aldrich) at a concentration of 1 mM. Si application was initiated simultaneously with drought imposition and supplied through the irrigation water used to maintain the designated field capacity levels throughout the treatment period, thereby ensuring continuous root-zone availability of Si. Drought treatments were imposed 14 days after seedling emergence by maintaining soil moisture at the designated FC levels. Soil moisture was monitored gravimetrically through daily weighing and adjusted as required to maintain assigned FC treatments throughout the experimental period. The drought and Si treatments were continued for 26 days, after which four biological replicates from each treatment were harvested for physiological, biochemical and molecular analyses. The remaining plants were maintained under their respective treatment conditions until 90% maturity for the assessment of reproductive and yield-related traits. Field capacity was determined gravimetrically. A 100 g sample of air-dried soil was placed in a container and water was added gradually while mixing until the soil reached a uniformly saturated paste without free drainage. The soil reached saturation at 100 mL of water, which was taken as 100% field capacity. Treatment levels (75, 50, and 25% FC) were calculated based on this value.

### Morphological and yield traits measurements

Data on different traits were recorded throughout the growth period and at maturity. Measurements included plant height (PH), leaf length (LL), leaf width (LW), Shoot Length (SL), Root Length (RL), and pod length (PDL), all measured in centimeter (cm) using a measuring scale. LL and LW were determined using the third and fourth youngest, fully expanded leaves from the top of the main stem on each plant. Moreover, 100 seed weight (100 SW), root fresh weight (RFW), root dry weight (RDW), shoot fresh weight (SFW) and shoot dry weight (SDW) were measured using an electrical weighing balance. Dry weights were obtained after oven drying at 70 °C for 72 h to constant weight. Leaf area (LA) was calculated by multiplying LL and LW. Data for other traits such as number of leaves per plant (NOL/P), days to 50% flowering (DTF 50%), days to 75% flowering (DTF 75%), days to 90% flowering (DTF 90%), days to 50% maturity (DM 50%), days to 75% maturity (DM 75%), days to 90% maturity (DM 90%), number of pods per plant (NOP/P), number of clusters per plant (NOCP) and number of pods per cluster (NOPC) were also measured.

### Physiological and plant water relations measurements

Chlorophyll contents was measured by grinding 0.1 g of fresh leaf, grinding, homogenized in 80% methanol and storing at 4 °C overnight. Absorbance at different wavelengths (480, 645, and 663 nm) was measured using a spectrophotometer. Chlorophyll concentrations were calculated using the following formula:

Chlorophyll *a* (mg g^−1^ f.wt) = [12.7 (OD 663) – 2.69 (OD 645)] × V/(1000) × W

Chlorophyll *b* (mg g^−1^ f.wt) = [22.9 (OD645) – 4.68 (OD663)] × V/(1000) × W

V = volume of the extract (ml)

W = weight of fresh leaf tissue (g)

Carotenoid (Car) content was measured using 0.1 g of fresh leaf tissue homogenized in 80% methanol. The homogenate was centrifuged at 10,000 × g for 15 min and the resulting supernatant was used to measure carotenoid content at 480 nm using spectrophotometer. Relative water content (RWC%) was determined for fully expanded leaves using the formula: RWC (%) = [(Fresh Weight – Dry Weight)/(Turgid weight – Dry Weight)] × 100^24^. Osmotic potential (Ѱπ) was determined using fourth fully expanded leaf from each plant in each treatment using a freezing-point depression osmometer, following^25^. Water potential (Ѱω) was assessed using pressure chamber technique. Mean osmotic potential and water potential were used to estimate pressure potential (Ψp) using formula (Ψp = Ѱω – Ѱπ).

### Antioxidant enzymes, osmolytes and phenolic compounds

Antioxidant enzymes activities, including ascorbate peroxidase (APX), catalase (CAT), peroxidase (POD), superoxide dismutase (SOD), Malondialdehyde (MDA), total soluble proteins (TSP) and proline content, were determined in young plant leaves. 0.5 g leaf tissue from each plant and treatment was homogenized in phosphate buffer solution (1 mL). For each enzyme assay, 50 µL of homogenate was used.

#### Enzymatic antioxidants

SOD activity was determined through reaction mixture contained distilled water (400 µL), Triton X-100 (µL), methionine (100 µL), potassium phosphate buffer (50 µL), riboflavin (50 µL), NBT (50) µL and homogenate (50) µL. The prepared mixture was kept under intense light for 15 min to produce lamp and complete the reaction. The absorbance was then measured at 560 nm using spectrophotometer. POD activity was measured by preparing a mixture containing 100 µL hydrogen peroxide, 100 µL guaiacol, 700 µL potassium phosphate buffer and 50 µL homogenate. The absorbance was measured at different time intervals (0, 20, 40, 60, 80, 100 and 120 s) at 470 nm using spectrophotometer. CAT activity was estimated by preparing a mixture containing 0.1 mL enzyme extract, 2.8 mL phosphate buffer, and 1 mL hydrogen peroxide. The absorbance was recorded at 249 nm using a spectrophotometer following the method described by^26^. TSP were measured using 500 mg leaf tissue, grounded in 10 mL buffer and centrifuged at 11,000 rpm for 10 min at 4 °C. The resulting 100 µL of supernatant was mixed with 2 mL Bradford reagent and incubated for 15 min. The readings were then recorded at 595 nm using the^27^ method. APX and proline content were measured following the protocols (with some modifications) described^28,29^.

#### Stress markers, osmolytes and non-enzymatic antioxidants

MDA content was measured by the method describe by Heath and Packer (1968)^30^. Phenolic compounds (gallic acid, caffeic acid, ferulic acid, quercetin and vanillic acid) were determined using 100 mg of freeze-dried leaf tissue. Samples were extracted with 1.5 ml of methanol, formic acid and water mixture (25:24:1, v/v/v). The mixture was sonicated for 1 h with vigorous vortexing every ten minutes. Later, the mixtures were incubated at 4 °C overnight. The extracts were then centrifuged for 15 min at 11,000 × g (at 4 °C), and the supernatant was filtered through a 0.22 μm membrane filter. Phenolic analyses were performed using an HPLC system at the Department of Chemistry, Government College University Faisalabad (GCUF).

### Gas exchange and chlorophyll fluorescence measurements

Net assimilation rate (A; µmol CO₂ m⁻^2^ s⁻^1^), intercellular CO₂ concentration (Ci; µmol mol⁻^1^), transpiration rate (E; mmol m⁻^2^ s⁻^1^), and stomatal conductance to water vapor (gsw; mol m⁻^2^ s⁻^1^) were measured using a portable infrared gas analyzer (IGRA). Measurements were taken on fully expanded, healthy leaves from the upper canopy. Gas exchange measurements were conducted between 09:00 and 11:00 h under controlled conditions to minimize diurnal variation. During measurements, photosynthetically active radiation (PAR) inside the leaf chamber was maintained at 1000 µmol photons m⁻^2^ s⁻^1^ using a red-blue light source. The reference CO₂ concentration was set at 400 µmol mol⁻^1^, leaf temperature was actively regulated and maintained at 25 ± 2 °C using a built-in thermoelectric (Peltier) system of the leaf chamber. Relative humidity within the chamber was kept between 60 and 70%. Leaves were allowed to equilibrate for 2–3 min before data recording. Electron transport rate (ETR) was determined simultaneously using the chlorophyll fluorescence module of the IRGA. The ratio of electron transport to CO₂ assimilation (ETR/A; µmol e⁻ (µmol CO₂)⁻^1^) was calculated to assess photosynthetic efficiency under different treatments. Water use efficiency (WUE) was calculated as the ratio of net assimilation rate to transpiration rate using the formula (WUE = A/E), where A is net CO₂ assimilation (µmol CO₂ m⁻^2^ s⁻^1^) and E is transpiration rate (mmol H₂O m⁻^2^ s⁻^1^). For each treatment, measurements were taken from three biological replicates, and mean values were used for statistical analysis.

### Determination of silicon and mineral nutrients

Leaf samples were collected at vegetative stage after 26 days of treatment. Fully expanded leaves were washed sequentially with tap water, 0.1% HCl, and distilled water to remove surface contaminants. Samples were oven-dried at 70 °C for 72 h until constant weight, then ground into a fine powder using a stainless-steel grinder. 0.5 g of dried leaf powder was digested using a di-acid mixture of concentrated nitric acid (HNO₃) and perchloric acid (HClO₄) in a ratio of 3:1 (v/v). The digestion was carried out on a hot plate at 180 °C until a clear solution was obtained. After cooling, the digest was filtered and diluted to a final volume of 50 mL with deionized water. Calcium (Ca), magnesium (Mg), iron (Fe), manganese (Mn), zinc (Zn), copper (Cu), and boron (B) were quantified using atomic absorption spectrophotometry^31^. Phosphorus (P) was determined colorimetrically using the vanadomolybdate method and measured at 420 nm using a UV–Vis spectrophotometer (Gupta et al. 1993). Potassium (K) was determined using flame photometry. Dried and powdered leaf and root samples were digested using alkaline digestion (NaOH method). Silicon concentration was determined using molybdenum blue colorimetric method at 650 nm, following^32^.

### RNA extraction, cDNA synthesis and real-time quantitative PCR (RT-qPCR)

*DREB2A*, *PIP2-1*, and *TIP4-1* were selected based on their established roles in drought stress adaptation and plant water transport. *DREB2A* is a well-characterized drought-responsive transcription factor that regulates the expression of numerous stress-inducible genes involved in abiotic stress tolerance. The aquaporin genes *PIP2-1* (Plasma Membrane Intrinsic Protein 2–1) and *TIP4-1* (Tonoplast Intrinsic Protein 4–1) were selected because they represent two major aquaporin subfamilies involved in cellular and transcellular water movement, membrane hydraulic conductivity, and maintenance of plant water status under drought conditions. Evaluating the expression of these genes enabled investigation of the molecular basis of silicon-mediated regulation of water transport and drought adaptation in *Vigna mungo*. To study the expression of drought responsive transcription factor *DREB2A* and aquaporin associated genes (*PIP2-1 and TIP4-1*), leaf tissues which stored at − 80 °C were taken and grounded into fine powder using liquid nitrogen. From the grounded powder, 50 mg was used to extract total RNA using Thermo Scientific™ GeneJET RNA Purification Kit, following the manufacturer’s instructions. RNA concentration and purity were assessed using Thermo NanoDrop 2000 (Thermo Fisher Scientific, Waltham, MA, USA). cDNA was synthesized from 1 µg of total RNA using Alli-in-One Thermo Fisher Scientific (Waltham, MA, USA) First-Standard cDNA synthesis kit. The synthesized cDNA was stored at − 20 °C until further analysis. qRT-PRC was performed using CFX96 Touch™ Real-Time PCR Detection System (Bio-Rad, USA) with Universal SYBR Green iTaq SuperMix. Gene-specific primers (Table S1) were designed using the online QuantPrimer tool (https://quantprime.mpimp-golm.mpg.de/). Gene sequences associated with drought tolerance (*DREB2A*) and aquaporins (*PIP2-1 and TIP4-1*) in mung bean previously reported by^33,34^, were retrieved from the NCBI database. Full-length coding sequences of the target gene were downloaded in FASTA format and used as queries in BLASTn search against *Vigna mungo* nucleotide database at NCBI to identify orthologous sequences. BLASTn was performed using default parameters, and sequences showing the highest similarity (≥ 80–85% sequence identity), maximum query coverage, and the lowest E-value were selected as putative orthologs. The identified *Vigna mungo* orthologs were aligned with the corresponding mung bean gene sequences using Clustal Omega to confirm sequence conservation and to identify conserved regions suitable for primer design. Each RT-qPCR was performed using three biological replicates and three technical replicates per treatment. Relative gene expression levels were normalized using *Actin* and *elongation factor-1 alpha (EF-1α)* as internal reference genes. Relative transcript abundance calculated using the 2^–ΔΔCt^ method described by^35^.

### Soil analysis

Soil analysis was performed prior to filling the plastic pots and sowing to determine soil physiochemical properties and nutrient status required for plant growth and development. The soil pH and electrical conductivity (EC) were measured at 7.18 and 1.2 dS m⁻^1^, respectively, using a digital pH meter and a conductivity meter. The soil contained 0.074% nitrogen (equivalent to 740 mg/kg), available phosphorus 7.23 ppm, potassium 162 ppm and organic matter 1.75%. The Kjeldahl method was used to determine total nitrogen, the Olsen method to determine available phosphorus and flame photometry used to determine exchangeable potassium. The Walkley Black method was used to determine organic matter content.

### Statistical analysis

The data recorded for morphological, physiological, biochemical and molecular parameters were subjected to a two-way analysis of variance (ANOVA) to assess the main effects of genotype, treatments and their interaction. Prior to analysis, data was checked for normality and homogeneity of variance. Mean comparisons were performed using Tukey’s honestly significant difference (HSD) test at *P* ≤ 0.05. A Pearson correlation analysis was performed to evaluate the relationship among the morphological, physiological, biochemical, and molecular traits. To visualize interrelationships among traits, hierarchical clustering heatmaps were generated using the *corrplot* and *pheatmap* packages, providing a graphical representation of positive and negative associations among variables. Principal component analysis (PCA) was performed to identify key traits contributing to variability among treatments and genotypes and to visualize treatment-wise clustering of combined physiological, biochemical, and molecular responses. All the statistical analyses were performed using R (version 4.5.1) software. ANOVA was performed using the *agricolae* packages; PCA was performed using the *factoextra* and *FactoMineR* packages; correlation analyses were performed using *corrplot* package. Structural Equation Modeling (SEM) was performed to examine direct and indirect relationships among silicon accumulation, growth traits, physiological attributes, antioxidant activities, and gene expression variables. SEM analyses were conducted separately for each genotype (Chakwal and Arooj) using the *lavaan* package. Latent constructs were developed to represent functional trait groups (Growth, Biochemical responses, Physiological responses, Gene expression, and Silicon accumulation). Model estimation was performed using maximum likelihood estimation. Model fit was evaluated using standard indices including χ^2^ test statistics, Comparative Fit Index (CFI), Tucker–Lewis Index (TLI), Root Mean Square Error of Approximation (RMSEA), and Standardized Root Mean Square Residual (SRMR). Graphical representation of SEM path diagrams was generated using the *tidySEM* package, allowing visualization of standardized path coefficients and structural relationships among latent and observed variables. All the bar graphs were generated and statistical analyses were performed using RStudio.

## Results

### Analysis of variance

Two-way ANOVA revealed that genotype, treatments, and their interaction differentially effected morphological, physiological, biochemical, mineral and molecular traits (Table S2). Genotypes and treatments exerted highly significant effects (*P* ≤ 0.01 and *P* ≤ 0.001) on all vegetative and phenological parameters, including PH, LL, LW, LA, SL, RL, DTF (DF50, DF75, DF90, DM50, DM75, DM90), PDL, NOP/P, 100 SW, RFW, RDW, SFW, SDW, Chl *a*, Chl *b*, and Car. The magnitude of genotype effects was particularly strong for maturity traits (e.g., DM90, F = 1917***). The genotype × treatment interaction was significant for PH, LL, LW, SL, DF50, DF75, DM50, DM75, DM90, SDW, Chl *b* and Car indicating differential responses to drought and Si supplementation. While this interaction was non-significant for LA, RL, NOL/P, DF90, PDL, 100 SW, NOCP, NOPC, RFW, RDW, SFW, and Chl a, indicating trait-specific genotype sensitivity under combined drought and silicon treatments.

Antioxidant enzyme activities (SOD, POD, CAT, APX, MDA), TSP, RWC, osmotic potential (OP), pressure potential (PP), and stomatal conductance (SO) and net assimilation rate (NAR), were significantly affected by both genotype and treatment. Whereas, water potential (WP), NAR, intercellular CO_2_ concentration (Ci) and transpiration rate (TR) were not significantly influenced by genotypes. The genotype × treatment interactions were largely non-significant for antioxidant and gas exchange traits, except for water potential, indicating that most biochemical and physiological responses to drought and Si were consistent across genotypes. Similarly, mineral nutrients (K, Ca, Mg, P, S, B, Fe, Mn, Zn) and WUA were significantly influenced by genotype and treatment, whereas Cu was not significantly affected by genotype. Moreover, P (F = 190.5***) and Zn (F = 66.5***) showed particularly strong responses to treatments. Significant genotype × treatment interactions were detected for Mg, P, Fe, Mn, Zn, gallic acid and silicon concentrations in leaves and roots, suggesting cultivar-specific nutrient modulation under drought and Si combination.

Both genotype and treatment significantly (*P* ≤ 0.001) influenced phenolic compounds including caffeic acid, ferulic acid, quercetin and vanillic acid. Interaction effects were also significant for all phenolics, indicating different metabolic adjustment between cultivars studied. The expression of drought response gene DREB2A (key transcription factor) and aquaporin genes (*PIP2-1* and *TIP4-1*) was also significantly affected by genotypes, treatment and their interaction (*P* ≤ 0.01 and *P* ≤ 0.001). This demonstrates a genotype-dependent transcriptional regulation under drought and Si supplementation.

### Drought stress reduced vegetative growth, biomass and reproductive traits

**Fig. 1:**
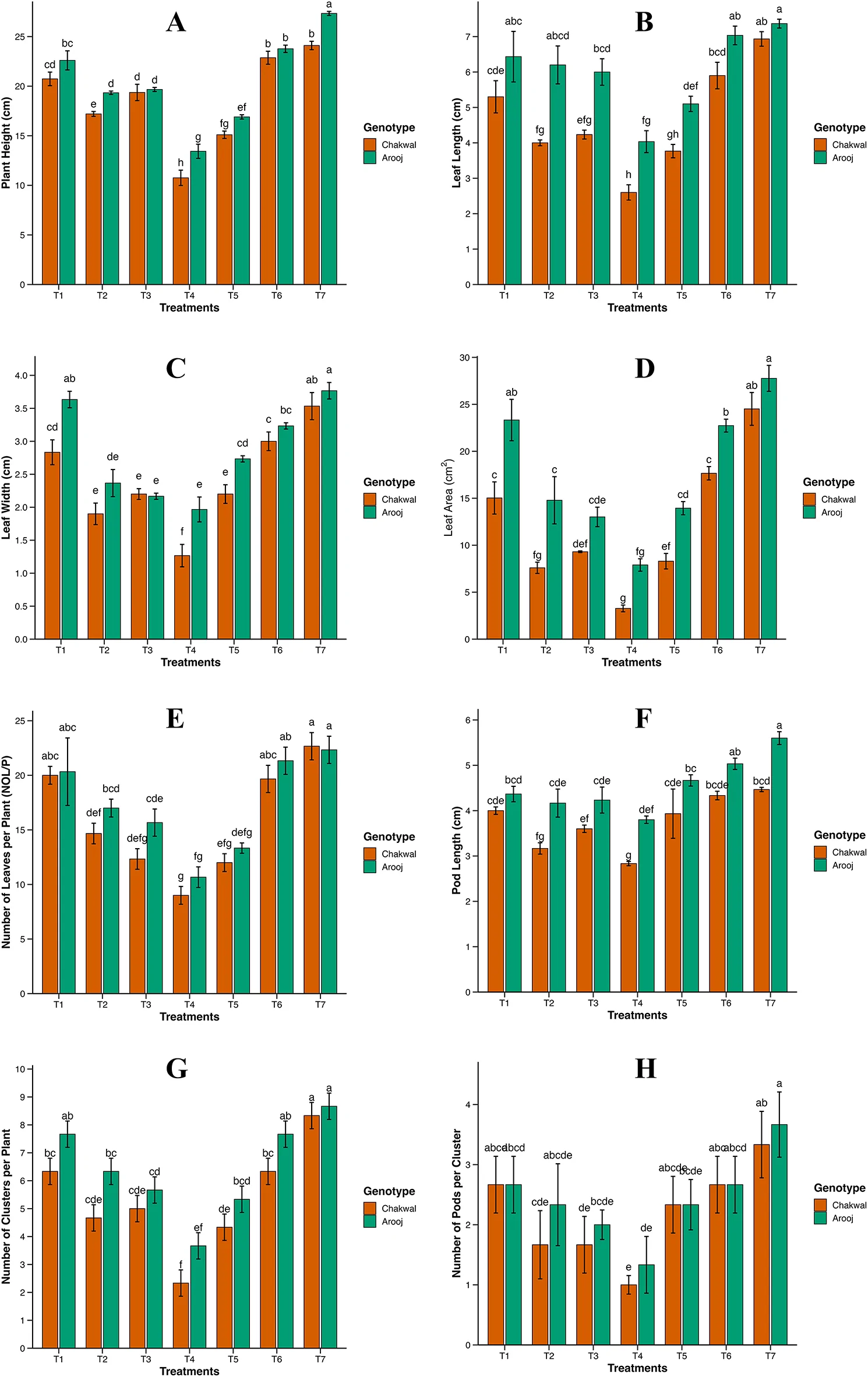
Effect of drought stress and Si supplementation on different vegetative and reproductive traits. Data are presented as mean ± SE and SD. Different letters indicate significant differences among treatments according to Tukey’s HSD test. Treatments were as follow: T1 (controlled: 100% FC), T2 (75% FC), T3 (50% FC), T4 (25% FC), T5 (25% FC + Si), T6 (50% FC + Si) and T7 (75% FC + Si).

**Fig. 2:**
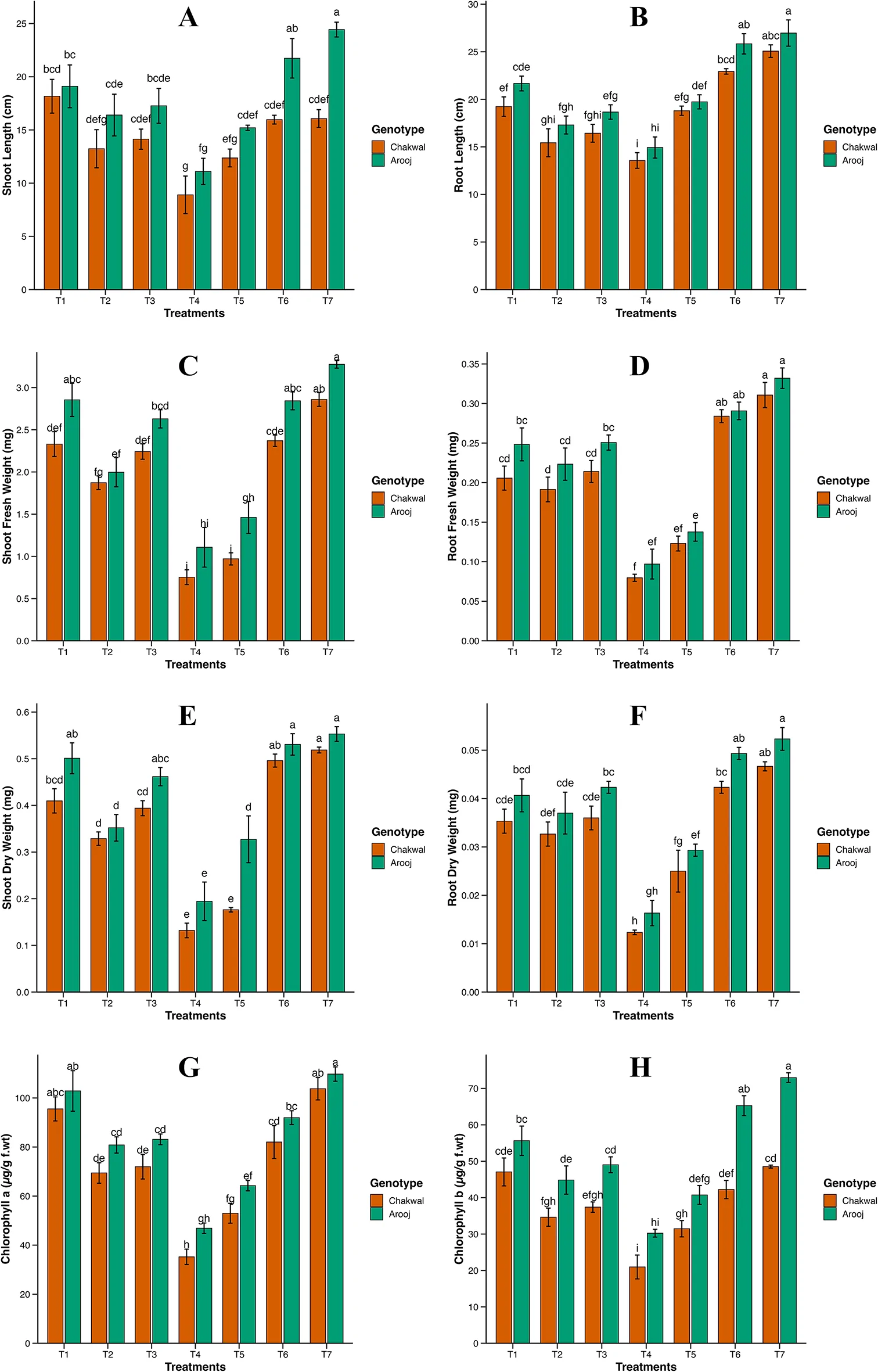
Effect of drought stress and Si supplementation on different biomass and chlorophyll traits. Data are presented as mean ± SE and SD. Different letters indicate significant differences among treatments according to Tukey’s HSD test. Treatments were as follow: T1 (controlled: 100% FC), T2 (75% FC), T3 (50% FC), T4 (25% FC), T5 (25% FC + Si), T6 (50% FC + Si) and T7 (75% FC + Si).

Drought stress significantly inhibited vegetative growth in both *Vigna mungo* cultivars, with the magnitude of reduction increasing as soil moisture decreased. Severe drought (25% FC) caused the greatest decline in plant height leaf dimensions, leaf area, leaf numbers, shoot length and root length compared with the well-watered control (Fig. 1A–E; Fig. 2A,B). Although both cultivars were negatively affected, Chakwal generally maintained better growth performance under water deficit than Arooj. Si supplementation significantly alleviated the adverse effects of drought and improved all measured vegetative growth traits, particularly under severe drought conditions, indicating a protective role of Si in sustaining plant growth under water limitation.

A similar trend was observed for biomass accumulation. Drought stress markedly reduced shoot and root fresh and dry weights in both cultivars, with the largest reduction occurring under 25% FC (Fig. 2C–F). The decline in biomass reflected the strong inhibitory effect of water deficit on plant growth and resource allocation. Si supplementation substantially improved biomass production under drought stress, resulting in higher shoot and root fresh and dry weights compared with non-supplemented drought stressed plants. The beneficial effects of Si were particularly evident under moderate and severe drought treatments, demonstrating its capacity to partially restore biomass accumulation under water-deficit conditions.

**Fig. 3:**
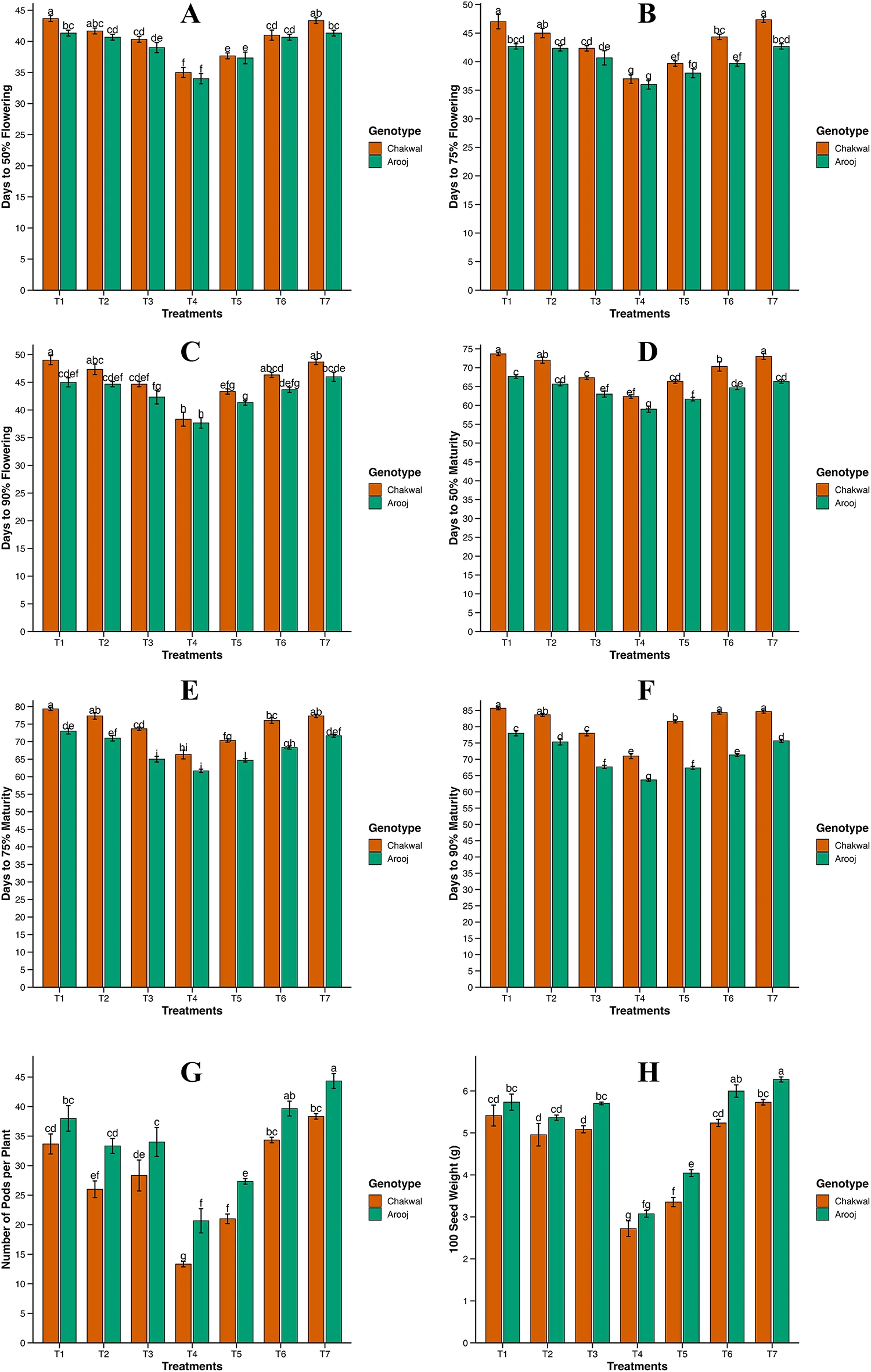
Effect of drought stress and Si supplementation on different reproductive traits. Data are presented as mean ± SE and SD. Different letters indicate significant differences among treatments according to Tukey’s HSD test. Treatments were as follow: T1 (controlled: 100% FC), T2 (75% FC), T3 (50% FC), T4 (25% FC), T5 (25% FC + Si), T6 (50% FC + Si) and T7 (75% FC + Si).

Reproductive development was also highly sensitive to drought stress. Water deficit accelerated flowering maturity, resulting in earlier reproductive transition in both cultivars (Fig. 3A–F). In addition, drought significantly reduced yield-related traits, including the number of pods pe plant, clusters per plant, pod length, and 100 seed weight, with the greatest reductions observed under severe drought stress (Fig. 3G–H). Si supplementation mitigated these detrimental effects and improved reproductive performance under drought conditions. Si treated plants exhibited delayed flowering and maturity relative to drought stressed plants without Si, suggesting improved physiological status and prolonged growth duration. Furthermore, Si significantly increased pods production, cluster formation, pod development, and seed weight under drought stress. Overall, the results demonstrate that Si application effectively counteracted drought-induced reductions in growth, biomass accumulation, and reproductive performance in both cultivars, with more pronounced benefits under moderate and severe drought conditions.

### Physiological, water relations and osmotic adjustment

**Fig. 4:**
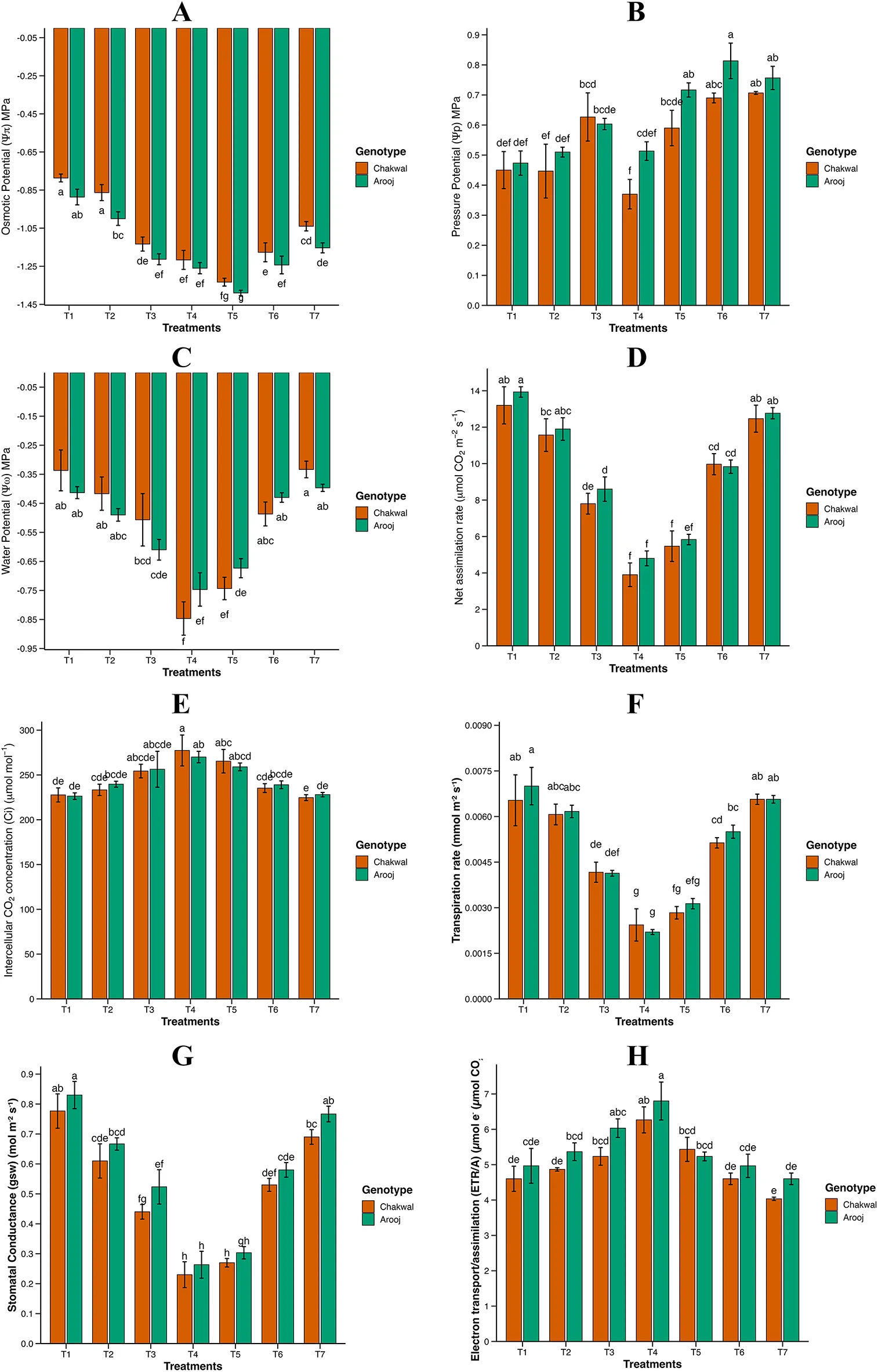
Effect of drought stress and Si supplementation on different osmotic and leaf gas exchange traits. Data are presented as mean ± SE and SD. Different letters indicate significant differences among treatments according to Tukey’s HSD test. Treatments were as follow: T1 (controlled: 100% FC), T2 (75% FC), T3 (50% FC), T4 (25% FC), T5 (25% FC + Si), T6 (50% FC + Si) and T7 (75% FC + Si).

**Fig. 5:**
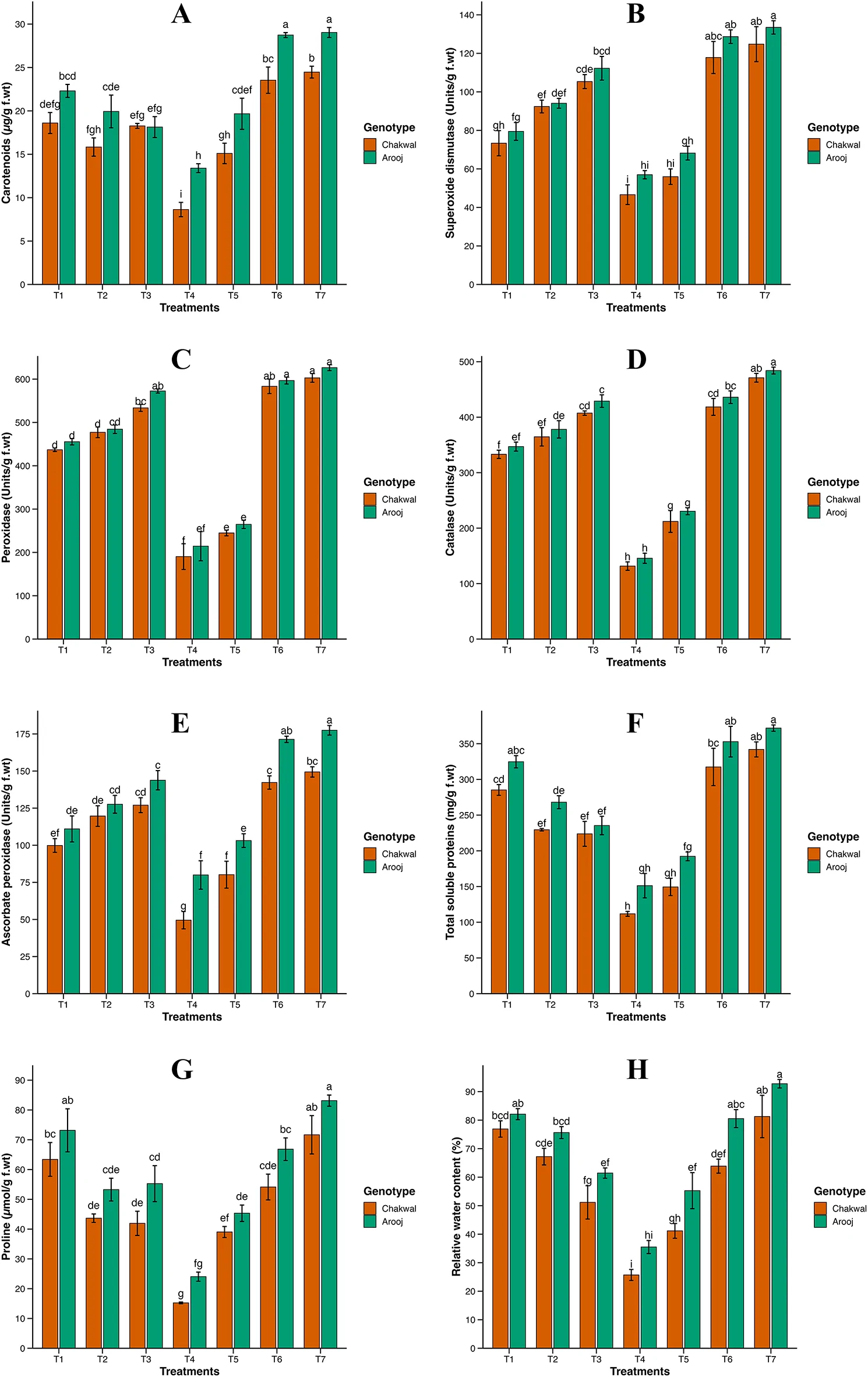
Effect of drought stress and Si supplementation on different antioxidant enzyme activities. Data are presented as mean ± SE and SD. Different letters indicate significant differences among treatments according to Tukey’s HSD test. Treatments were as follow: T1 (controlled: 100% FC), T2 (75% FC), T3 (50% FC), T4 (25% FC), T5 (25% FC + Si), T6 (50% FC + Si) and T7 (75% FC + Si).

Drought stress significantly affected photosynthetic pigments, water relations and osmotic attributes in both cultivars. Chlorophyll *a* content declined progressively with increasing drought intensity. In Chakwal, reductions of 27%, 24% and 63% were observed at 75% 50% and 25% FC, respectively, compared with the control (Fig. 2G). A similar trend was observed for chlorophyll *b*. Under 25% FC, chlorophyll *b* content reduced by 55% in Chakwal and 45% in Arooj relative to control, while reductions of 26% and 19% were recorded under mild stress 75% FC in Chakwal and Arooj, respectively (Fig. 2H). Carotenoids content was also adversely affected by drought stress. In Chakwal, carotenoid levels decreased by 15%, 2%, and 53% at 75%, 50% and 25% FC, respectively (Fig. 5A). In Arooj, the corresponding decreases were 10%, 18%, and 40% under the same treatments. Water relation parameters were markedly influenced by soil moisture deficit. RWC reduced significantly with increasing drought severity, with the greatest reduction observed at 25% FC in both cultivars (Fig. 5H). Si supplementation improved RWC under drought conditions. In Arooj, RWC values under T6 (50% FC + Si) and T7 (75% FC + Si) exceeded those observed in the control treatment. Osmotic potential also declined progressively with increasing drought stress. Under control conditions, both genotypes exhibited the highest (less negative) osmotic potential, with Chakwal (− 0.78 MPa) showing a slightly higher value than Arooj (− 0.88 MPa). Increasing drought stress from 75% FC to 25% FC resulted in a significant decline in osmotic potential in both cultivars, with the lowest values observed at 25% FC (Fig. 4A). Si supplementation further enhanced osmotic adjustment across all stress levels (T5–T7), with the more negative osmotic potential values observed in Si treated plants compared with corresponding drought only treatments. Water potential showed a similar pattern, decreasing progressive with increasing drought stress. Si supplementation mitigated the adverse effects of severe drought, particularly T5 (25% FC + Si), where water potential values showed partial recovery compared with T4. Across most treatments, Chakwal maintained relatively higher (less negative) water potential than Arooj, with the maximum value observed under T7 (Fig. 4C). Leaf pressure potential also declined significantly with increasing drought severity, reaching its lowest value at 25% FC in both cultivars. However, Si supplementation improved pressure potential under drought conditions. The highest recovery was recorded at 50% FC + Si, where Arooj exhibited high pressure potential (approximately 0.81). Across most treatments, Arooj maintained a relatively stronger pressure potential than Chakwal, particularly under Si-supplemented conditions.

### Antioxidant enzyme activities and phenolic compounds

The activities of antioxidant enzymes, including SOD, POD, CAT, MDA and APX, increased significantly with increasing drought stress. SOD activity increased by 26% and 43% at 75% and 50% FC, respectively, in Chakwal compared to the control (Fig. 5B). In Arooj, under the same treatments, it was increased by 18% and 41%. MDA content increased by 60% and 46% at 50% FC in Chakwal and Arooj, respectively, relative to control (Fig. 11D). Notably, at 75% FC, MDA levels rose by 30% in Chakwal and 10% in Arooj. Interestingly, at 25% FC, MDA levels were lower than the control, suggesting a severe growth suppression at this extreme deficit. Similarly, POD, CAT and APX activities increased under drought stress. POD activity increased by 22% and 25%, CAT activity by 22% and 24%, and APX activity by 27% and 31% in Chakwal and Arooj, respectively (Fig. 5C, 5D, 5E). The highest enzyme activities were recorded under 25% FC. All antioxidant enzymes reached their maximum levels in both cultivars. Si Supplementation further enhanced antioxidant enzyme activities under severe to moderate stress conditions (T5-T7). Compared with the corresponding drought-only treatments, Si-treated plants exhibited higher enzymes activities, with increases of approximately 41%, 29%, and 39% recorded for SOD, POD and APX, respectively. Si supplementation significantly mitigated oxidative damage by reducing MDA concentrations across all drought levels. Across treatments, Arooj generally maintained higher antioxidant enzymes activities than Chakwal under both drought and Si-supplemented conditions. TSP and proline accumulation varied significantly across cultivars and treatments (Fig. 5F, G). Progressive drought stress induced a substantial decline in TSP and proline levels. Under severe drought (25% FC) TSP and proline decreased by 68% and 76%, respectively, in Chakwal compared with control, whereas reductions of 53% and 67% were observed in Arooj. Si supplementation mitigated these reductions under drought conditions. At 25% FC + Si, TSP and proline contents increased by 34% and 156% in Chakwal and by 27% and 88% in Arooj, compared with the corresponding drought-only treatment. The most striking recovery was observed under 75% FC + Si, where TSP levels exceeded those recorded under control conditions.

**Fig. 6:**
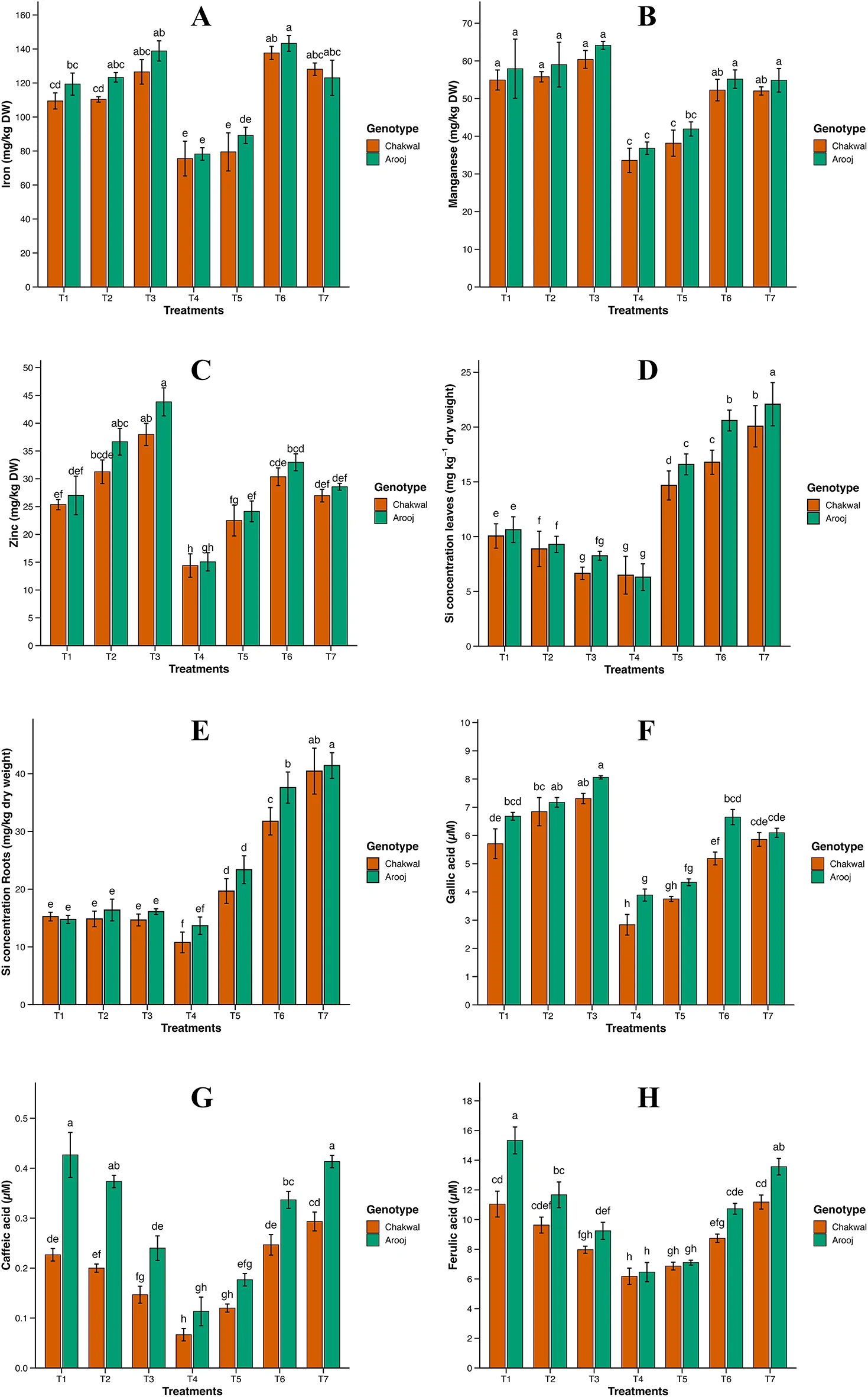
Effect of drought stress and Si supplementation on different mineral compositions and phenolic compounds. Data are presented as mean ± SE and SD. Different letters indicate significant differences among treatments according to Tukey’s HSD test. Treatments were as follow: T1 (controlled: 100% FC), T2 (75% FC), T3 (50% FC), T4 (25% FC), T5 (25% FC + Si), T6 (50% FC + Si) and T7 (75% FC + Si).

Phenolic compounds involved in secondary metabolism, including gallic acid, caffeic acid, ferulic acid, quercetin and vanillic acid were also influenced by drought stress and Si supplementation. The concentration of these compounds increased progressively with increasing drought severity from T1 to T4, and Si supplementation (T5-T7) further enhanced their accumulation. Gallic and vanillic acids reached their highest concentrations under 50% FC + Si. In Arooj, gallic acid and vanillic acid concentrations reached approximately 6.6 µM and 7.1 µM, respectively, representing substantial increases over the T4 (Figs. 6F, 9A). Quercetin content was highest under Si-treated drought conditions, particularly at T7, where Arooj maintained concentrations above 2.0 µM (Fig. 8A). Caffeic acid also increased under drought and Si treatments, with the highest levels observed at T7 in both cultivars, although overall concentrations remained relatively low (ranging from 0.2 to 0.4 µM) (Fig. 6G). Ferulic acid showed a robust response to Si supplementation, particularly in Arooj at T6 and T7 (Fig. 6H). While drought alone (T4) reduced ferulic acid compared to the control, Si supplementation resulted in a marked increase, with the highest concentration (approximately 13.5 µM) recorded in Arooj under T7.

### Drought altered and silicon modulated leaf gas exchange

**Fig. 7:**
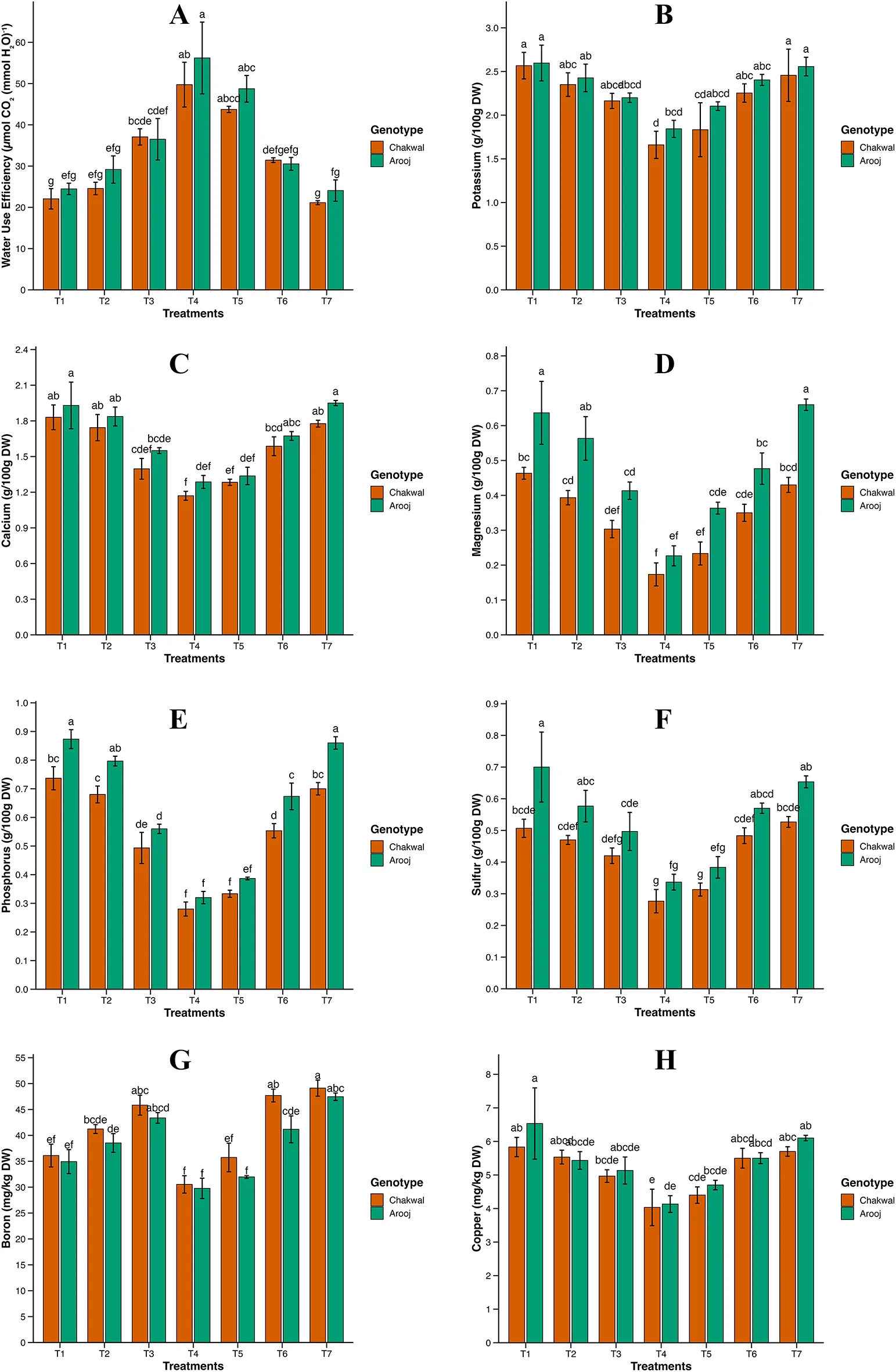
Effect of drought stress and Si supplementation on different mineral compositions and phenolic compounds. Data are presented as mean ± SE and SD. Different letters indicate significant differences among treatments according to Tukey’s HSD test. Treatments were as follow: T1 (controlled: 100% FC), T2 (75% FC), T3 (50% FC), T4 (25% FC), T5 (25% FC + Si), T6 (50% FC + Si) and T7 (75% FC + Si).

Drought stress significantly influenced gas exchange characteristics in both cultivars. Under control conditions, higher gas exchange rates were observed in both cultivars, with Arooj showing greater values than Chakwal. However, increasing drought intensity led to a progressive decline in net assimilation rate, transpiration rate and stomatal conductance. Under 25% FC, net assimilation rate declined to 3.9 µmol CO₂ m⁻^2^ s⁻^1^ in Chakwal and 4.8 µmol CO₂ m⁻^2^ s⁻^1^ in Arooj (Fig. 4D). Similarly, transpiration rate decreased to approximately 0.002 mmol m⁻^2^ s⁻^1^, while stomatal conductance declined to 0.23 and 0.26 mol m⁻^2^ s⁻^1^in Chakwal and Arooj, respectively (4F, G). These reductions were consistent with increasing drought severity across treatments. In contrast, intercellular CO_2_ concentration (277 and 270 µmol mol⁻^1^), electron transport/assimilation (6.2 and 6.8 µmol e⁻ (µmol CO₂)⁻^1^) and water use efficiency (49.7 and 56.2 µmol CO₂ (mmol H₂O)⁻^1^) increased under drought stress, reaching their highest values at 25% FC. Si supplementation significantly improved net assimilation rate, transpiration rate and stomatal conductance under water-deficit conditions. Meanwhile, intercellular CO_2_ concentration (Fig. 4E), electron transport/assimilation (Fig. 4H) and water use efficiency (Fig. 7A) decreased with Si supplementation, tending to stabilize toward values observed under control conditions.

### Impact of drought on mineral composition and silicon accumulation

The concentration of macro and micronutrients, including phosphorus, potassium, calcium, sulfur, magnesium, iron, manganese, zinc, copper, sulfur, boron, Si in leaves and roots, were analyzed at seedling stage under varying levels of drought stress and Si supplementation (Figs. 6,7). Drought stress significantly reduced the concentration of most mineral nutrients in both cultivars, whereas boron showed a contrasting trend with increase accumulation at 50 and 75% FC in both Chakwal and Arooj. The greatest reductions in nutrient concentration were recorded under 25% FC. Under this treatment, phosphorus decreased by 62 and 63%, potassium by 35 and 29%, calcium by 36 and 33%, magnesium by 62 and 65%, iron by 31 and 35%, zinc by 43 and 44%, and copper by 31 and 37% in Chakwal and Arooj, respectively, compared with control. Si supplementation significantly enhanced mineral accumulation under drought stress. At 25% FC + Si, potassium, calcium, magnesium, phosphorus, sulfur, copper, iron, manganese and zinc increased by 14, 9, 60, 21, 14, 13, 16, 14 and 60%, respectively, compared with 25% FC without Si. Si accumulation in leaves progressively reduced with the highest reduction observed at 25% FC, decreasing by 36% in Chakwal and 41% in Arooj compared with control (Fig. 6D). In contrast, the Si concentration in roots under drought stress remained largely unchanged, except for reduction at 25% FC in both cultivars (Fig. 6E). With the Si supplementation, the Si accumulation in both leaves and roots significantly increased by 126% and 83%, at 25% FC + Si in Chakwal, respectively, compared with 25% FC without Si.

### Regulation of key gene expression by drought stress and silicon

**Fig. 8:**
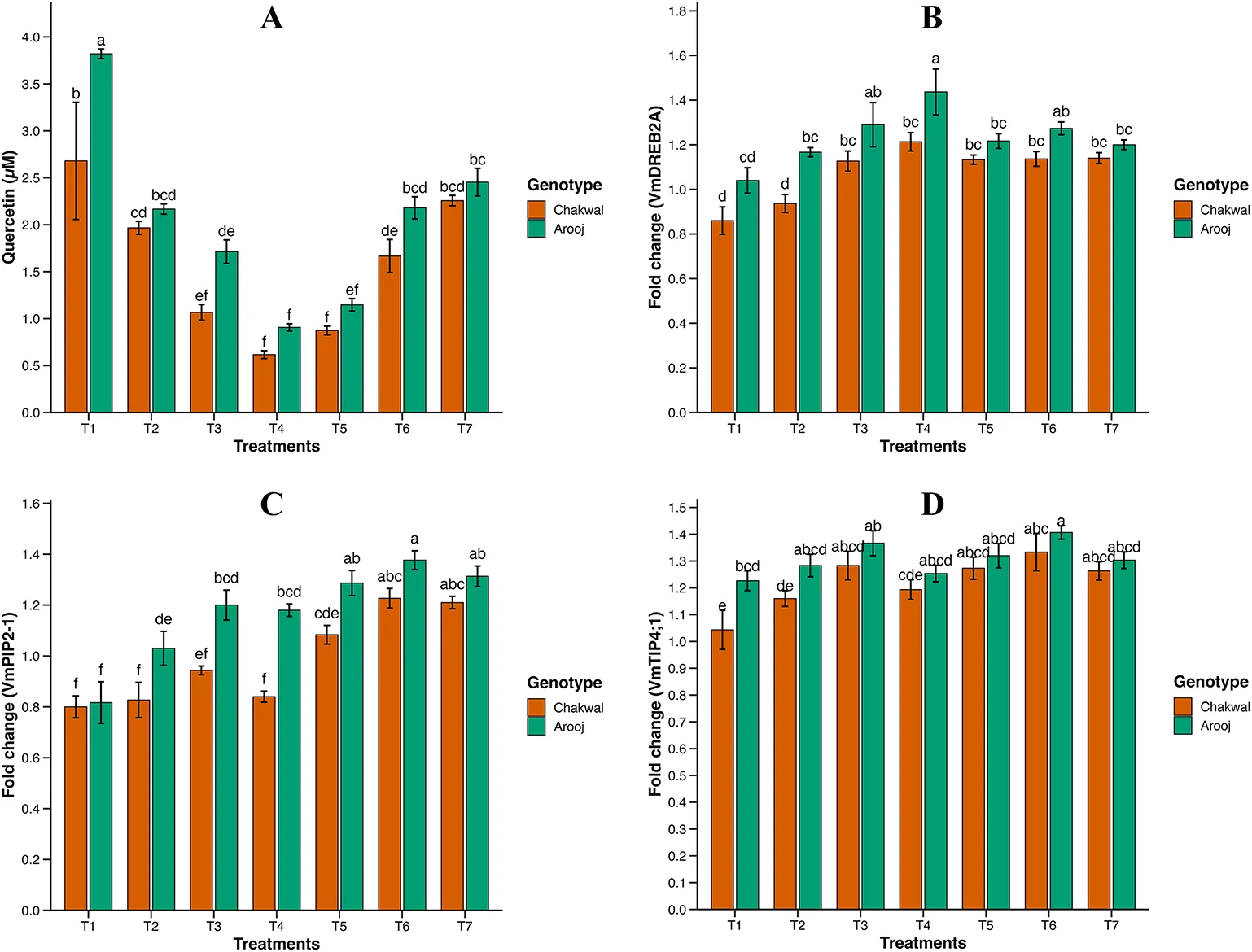
Effect of drought stress and Si supplementation on different gene expression. Data are presented as mean ± SE and SD. Different letters indicate significant differences among treatments according to Tukey’s HSD test. Treatments were as follow: T1 (controlled: 100% FC), T2 (75% FC), T3 (50% FC), T4 (25% FC), T5 (25% FC + Si), T6 (50% FC + Si) and T7 (75% FC + Si).

The expression levels of drought-responsive and aquaporin related genes, including *DREB2A*, *PIP2-1* and *TIP4-1* were evaluated to access transcriptional responses to drought stress and Si supplementation (Fig. 8B–D). Overall, progressive drought stress significantly upregulated the expression of all three genes in both cultivars, while Si supplementation further modulated their transcriptional responses. For *DREB2A*, Arooj exhibited slightly higher basal expression under control conditions (fold change ≈ 1.04) compared to Chakwal (≈ 0.86). As drought intensified, transcript abundance increased progressively in both cultivars, reaching maximum levels under 25% FC. Under this treatment, *DREB2A* expression reached approximately 1.44-fold in Arooj and 1.21-fold in Chakwal. Si supplementation maintained elevated transcript levels under drought conditions. Notably, at 25% FC + Si, expression levels in both cultivars remained significantly higher than those in the controls but slightly lower than those observed under drought stress alone. A similar trend was observed for *PIP2-1* expression. Under control conditions, both cultivars exhibited comparable baseline expression levels. As drought stress increased, a clear dose-dependent upregulation of *PIP2-1* was observed, with the highest expression under 25% FC. Under this treatment, Arooj showed significantly higher transcript abundance (≈ 1.18-fold) compared to Chakwal (≈ 0.84-fold). Si supplementation further enhanced *PIP2-1* expression across all drought levels, with the highest expression recorded in Arooj under 50% FC + Si, reaching approximately 1.38-fold compared with the corresponding drought treatment without Si. The same pattern of expression was observed under the same treatments for *TIP4-1*. At 50% FC, expression in Arooj reached a maximum fold change of approximately 1.37, which was higher than the corresponding level in Chakwal (1.28). Across all treatments, Arooj consistently exhibited higher transcript abundance of *DREB2A*, *PIP2-1* and *TIP4-1* compared with Chakwal, particularly under Si-supplemented conditions.

### Principal component analysis reveals distinct response patterns to drought stress and silicon treatments

**Fig. 9:**
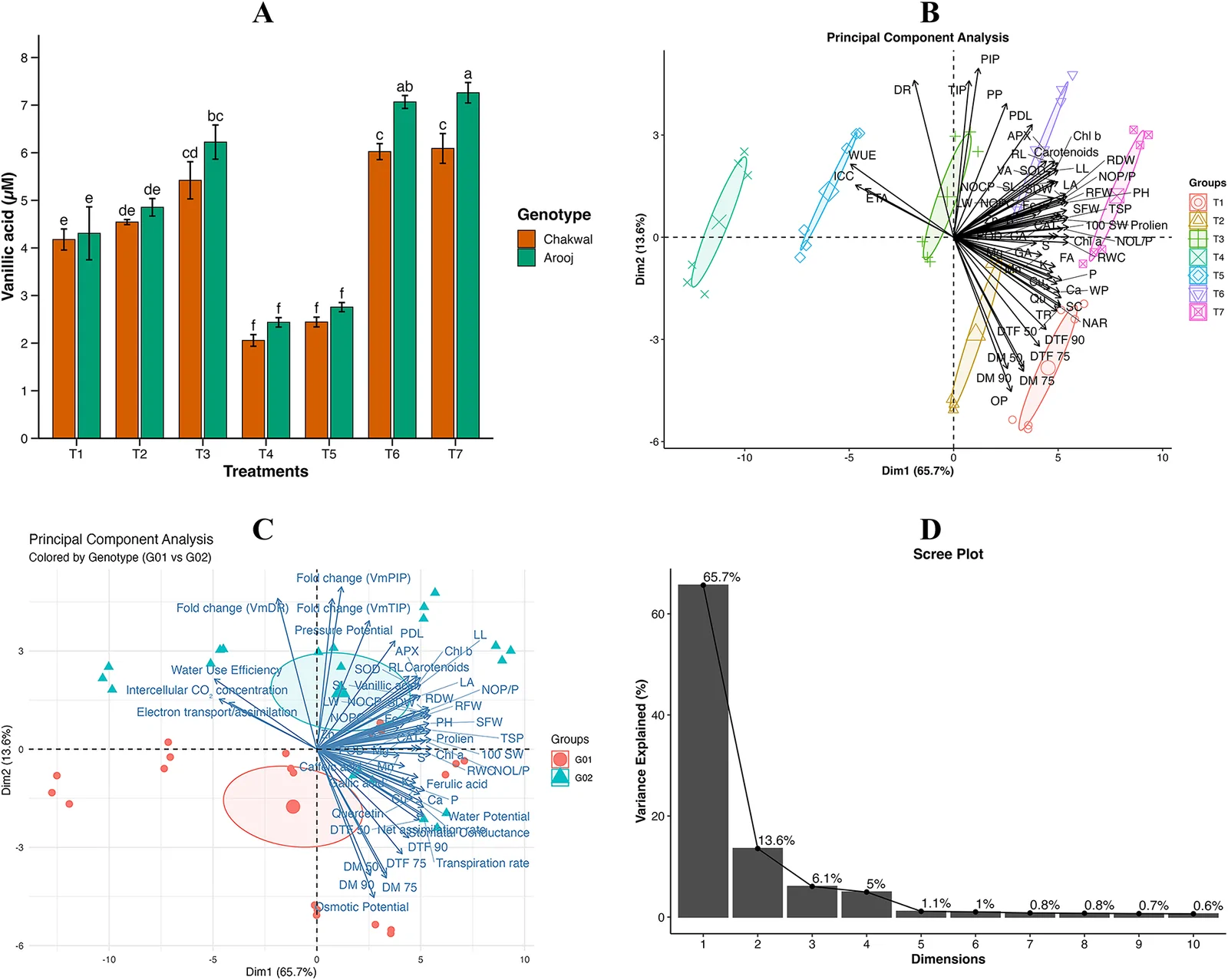
Effect of drought stress and Si supplementation on Principal Component Analysis. Data are presented as mean ± SE and SD. Different letters indicate significant differences among treatments according to Tukey’s HSD test. Treatments were as follow: T1 (controlled: 100% FC), T2 (75% FC), T3 (50% FC), T4 (25% FC), T5 (25% FC + Si), T6 (50% FC + Si) and T7 (75% FC + Si).

PCA was performed to integrate physiological, biochemical and molecular responses of both cultivars under drought stress and Si supplementation. The first two principal components (Dim1 and Dim2) explained 79.3% of the total variation, with Dim1 explaining 65.7% and Dim2 13.6% (Fig. 9B). The scree plot indicated a sharp decline in eigenvalues after the second component, confirming that these two dimensions captured most of the variability among treatments (Fig. 9D). The PCA biplot revealed clear separation among treatments, particularly highlighting the influence of Si. Control (T1) and Si supplemented treatments under moderate stress (T6 and T7) clustered on the positive side of Dim1. These groups showed a strong positive correlation with growth parameters (PH, RDW, RFW, LA), chlorophyll content (Chl *a*, Chl *b*), and TSP. In contrast, the severe drought treatment (T4) and its Si supplemented counterpart (T5) were positioned toward the negative side of Dim1. T4 was associated with higher values of WUE, Ci, and ETR/A, reflecting distinct physiological responses under severe moisture limitation. Interestingly, the expression of genes (*PIP2-1*, *TIP4-1*, and *DREB2A*) together and pressure potential showed strong positive loadings on Dim2 and were positioned close to Si-treated groups (T5 and T6). Biochemical variables, including antioxidant enzymes (SOD, CAT, APX) and phenolic compounds (gallic acid, ferulic acid, vanillic acid, quercetin), formed a tight cluster in the right-hand quadrants, showing strong correlations among these traits and their association with Si-treated plants.

Genotypic differentiation was primarily observed along Dim2. Arooj (G02) showed a positive association with gene expression variables and pressure potential, particularly under Si-supplemented regimes (T5 and T6). Conversely, Chakwal (G01) was more closely aligned with osmotic potential and certain phenolic compounds such as quercetin and gallic acid (Fig. 9C). These patterns highlight contrasting response strategies between the two cultivars under drought and silicon treatments.

### Pearson’s correlation and hierarchical clustering heatmap analysis

Pearson’s correlation analysis was performed to examine the relationships among the measured variables across different traits. Correlation matrices were generated separately for each treatment to assess treatment-specific associations. The systematic analysis of correlation matrices across all treatments (T1 to T4) and Si-supplemented treatments (T5 to T7) revealed a clear shift in the relationships among plants traits in response to drought stress and Si application (Fig. S1). As drought stress intensified from control to 25% FC, correlations between growth parameters (PH, LA, RFW, SDW) and reproductive traits (DTF, DM) shifted from weak to strongly negative (Fig. S1A-D). This indicates that severe drought was associated with reduced vegetative growth alongside accelerated reproductive developmental. Simultaneously, the positive correlation cluster among antioxidant enzymes and phenolic compounds suggests coordinated activation of enzymatic and non-enzymatic antioxidant systems as stress intensity increases. Across all treatments, a strong relationship was consistently observed between the expression of drought-responsive and aquaporin genes (*DREB2A*, *PIP2-1*, *TIP4;1*) and water status indicators (osmotic potential and water potential). As osmotic and water potentials became more negative with increasing drought severity, transcript abundance of these genes increased. In contrast, these genes showed a strong positive correlation with pressure potential and relative water content (Fig. S2), indicating their association with mechanisms that maintain cellular hydration and turgor under limited conditions. Si supplementation altered the correlation networks by strengthening positive associations between growth and metabolic parameters. In Si-supplemented plants, correlations between biomass traits and chlorophyll content (Chl *a*, *b*) were stronger compared with drought-only counterparts. Furthermore, Si application enhanced the association between WUE, ETR/A, and antioxidant activities, indicating improved coordination between photosynthetic rates oxidative stress mitigation under drought conditions. A distinct positive correlation cluster was also observed between essential mineral nutrients (K, Ca, Mg, P, S, Fe, Zn) and antioxidant defense components, particularly in Si-treated plants. This pattern suggests that enhanced nutrient accumulation was associated with increased antioxidant activity under Si-mediated stress mitigation.

**Fig. 10:**
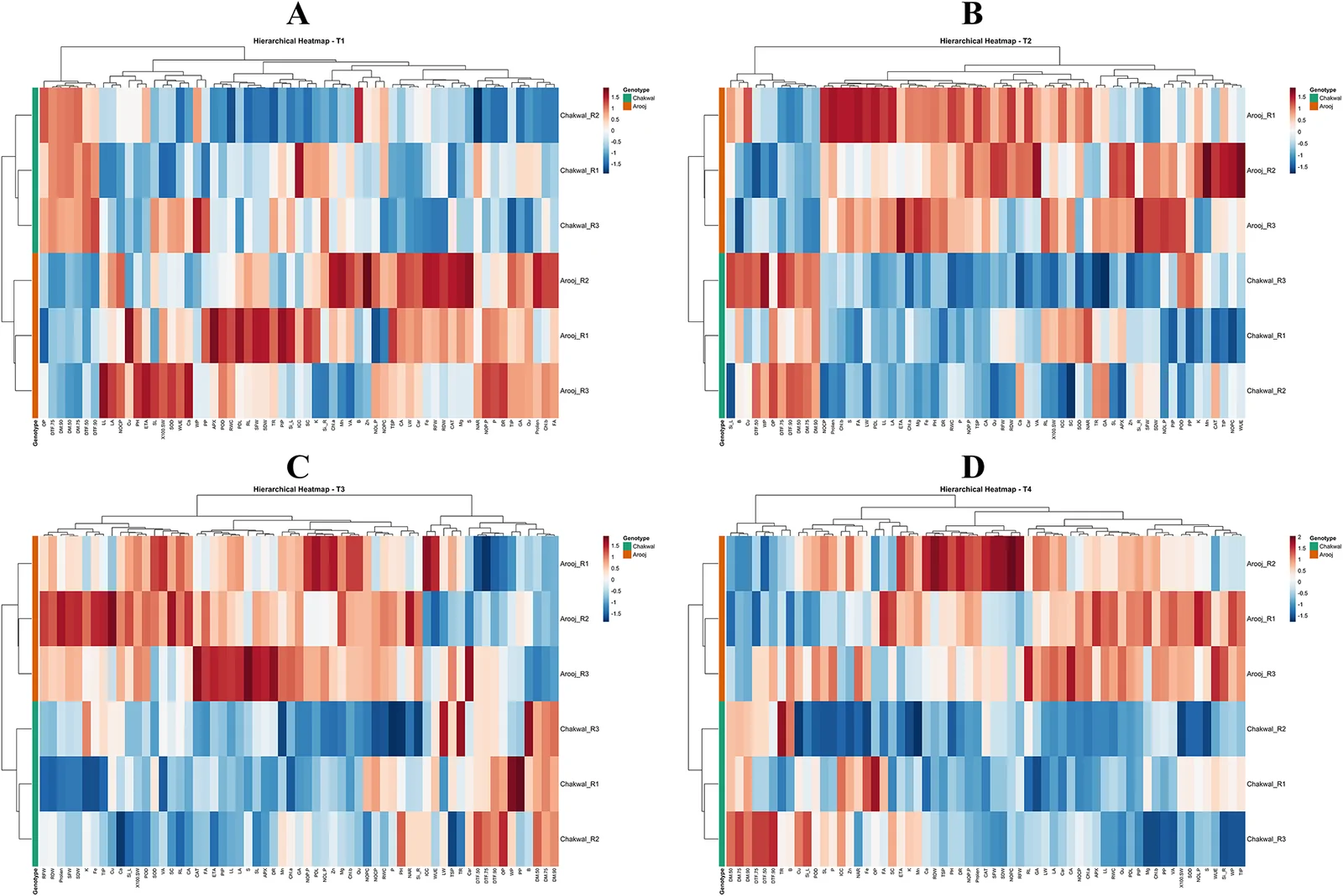
Hierarchical clustering heatmap showing relationships among morphological, physiological, biochemical, and molecular traits of two cultivars of *Vigna mungo* under different drought levels and silicon treatments. Color gradients represent relative trait variation across treatments, highlighting distinct clustering patterns associated with drought stress and Si-mediated responses.

**Fig. 11:**
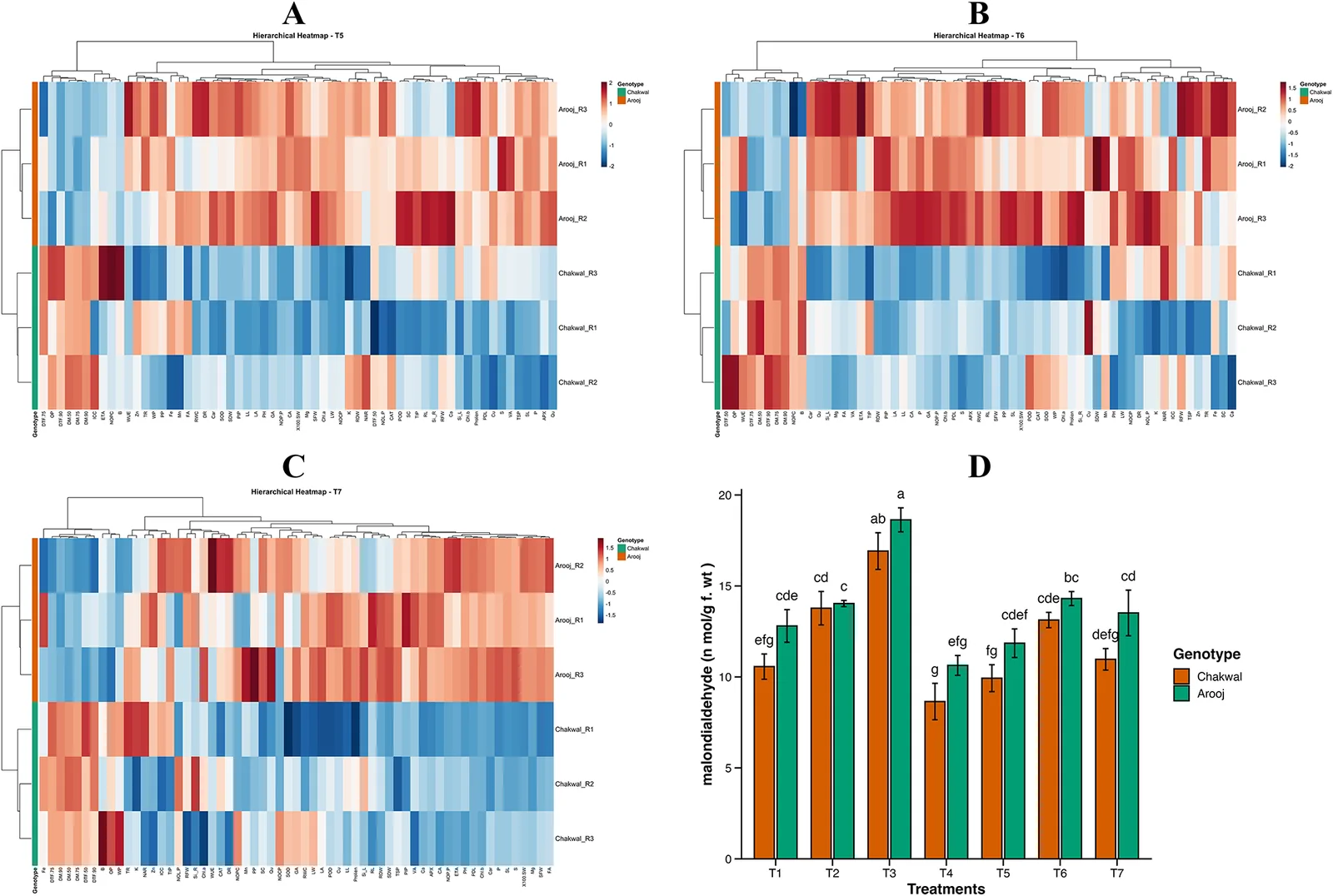
Hierarchical clustering heatmap showing relationships among morphological, physiological, biochemical, and molecular traits of two cultivars of *Vigna mungo* under different drought levels and silicon treatments. Color gradients represent relative trait variation across treatments, highlighting distinct clustering patterns associated with drought stress and Si-mediated responses.

Hierarchical clustering was further conducted to visualize genotype-specific responses across all measured traits. Under control conditions (T1), the primary vertical dendrogram clearly separated samples into two major clusters corresponding to the two genotypes. Arooj formed a cohesive cluster characterized by relatively higher values across growth parameters and antioxidant activities. In contrast, Chakwal exhibited comparatively lower levels for several traits (Fig. 10A). Morphological traits (PH, LL, LA, LW, NOL/P) and biomass parameters (RFW, SDW, 100 SW) formed a major cluster with stronger positive expression patterns in the Arooj, indicating greater vegetative vigor under non-stressed conditions. Antioxidant enzymes (SOD, CAT, POD, APX) and secondary metabolites (GA, VA, CA, FA, Qu) also clustered tightly together, with higher baseline activity in Arooj than Chakwal. The expression of stress-responsive genes (*DREB2A*, *PIP2-1*, *TIP4;1*) clustered with pressure potential, indicating a close association between molecular responses and plant water status (Fig. 10B). Under 50% FC (T3), developmental traits such as days to flowering (DTF) and days to maturity (DM) showed higher expression levels in Chakwal than in Arooj (Fig. 10C), suggesting an earlier reproductive transition in Chakwal under stress conditions. Under severe drought stress (25% FC), strong upregulation of antioxidant enzymes, phenolic compounds, and drought-responsive genes was observed, particularly in Arooj (Fig. 10D). In contrast, Chakwal showed reduced expression patterns across most growth and biomass traits, indicating stronger growth inhibition under severe drought. The heatmap for the T5 treatment (25% FC + Si) showed a clear ameliorative effect of silicon under severe drought stress. Si-treated plants showed improved expression of several physiological and biochemical traits in both genotypes. However, the response was more pronounced in Arooj (Fig. 11A). Si supplementation under moderate drought conditions (T6 to T7) further enhanced these responses (Fig. 11B). In particular, Arooj displayed increased expression of biomass related parameters, photosynthetic pigments, and nutrients (K, P, Mg, Ca), accompanied by higher pressure potential and relative water content (Fig. 11C). Chakwal also showed improvements in several phenolic compounds under Si supplementation; however, overall trait expression remained lower compared to Arooj. Overall, the hierarchical clustering across all seven treatments confirmed that Arooj possesses a more sensitive and efficient molecular framework for utilizing Si to maintain metabolic homeostasis and mitigate the detrimental effects of moisture deficit.

### Structural equation modeling

SEM provided a comprehensive framework for understanding the complex causal relationships among gene expression (G_E), biochemistry (Bch), physiological processes (Phy) and final yield (Yld) in both Chakwal and Arooj under drought stress and Si supplemented conditions. SEM is particularly useful in plant stress physiology because it simultaneously evaluates both direct and indirect pathways through which molecular, biochemical and physiological mechanisms influence crop productivity under abiotic stress. The present model revealed a hierarchical cascade in which gene expression regulates biochemical responses, which, in turn, modulate physiological performance and ultimately determine yield formation.

#### Gene expression and response

**Fig. 12:**
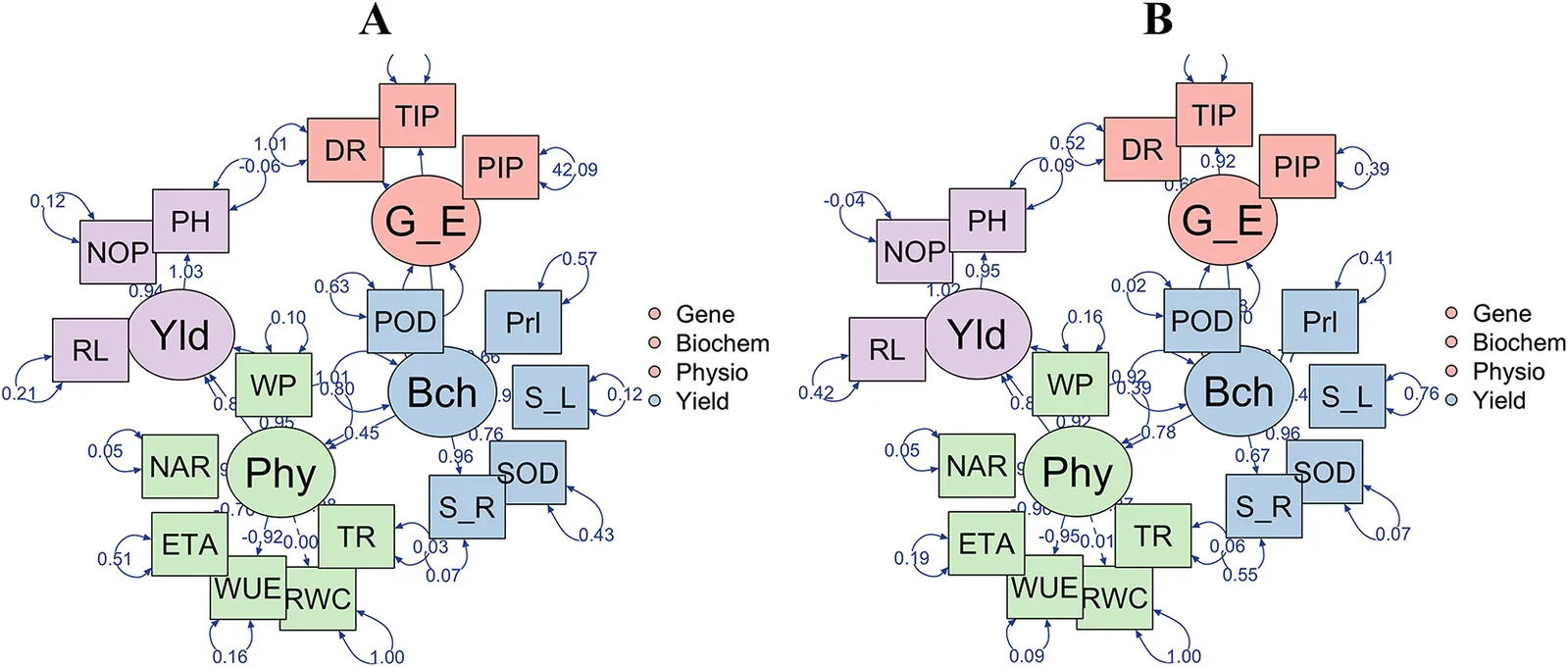
Structural equation modeling (SEM) illustrating the relationships among gene expression, biochemical traits, physiological parameters, and growth attributes in two cultivars of *Vigna mungo* under drought stress and silicon treatments. (**A**) SEM for Arooj and (**B**) SEM for Chakwal. Arrows indicate the direction of relationships among variables, while path coefficients represent the strength of direct effects.

Gene expression was presented by three important genes, including drought responsive transcription factor *DREB2A* and aquaporins *PIP2-1* and *TIP4-1.* Among these genes, TIP4-1 exhibited a strong positive loading of 0.92 on the gene expression construct, followed by *PIP2-1* (0.78) and *DREB2A* (0.69) (Fig. 12B). This indicated that aquaporins genes contributed more strongly to drought responsive transcriptional regulation than *DREB2A* in Chakwal. A similar pattern was observed in Arooj, where *TIP4-1* showed the strongest loading, followed by *PIP2-1* and *DREB2A*. Aquaporin genes regulate cellular water transport across membranes and play a critical role in maintaining cellular water homeostasis during drought stress. Enhancing the expression of these gene has likely improved hydraulic conductivity and cellular water balance, particularly with the availability of supplemented Si.

#### Influence of gene expression on biochemical adjustments

SEM analysis revealed a very strong positive relationship between gene expression and biochemical responses (GE → Bch = 0.92) in Chakwal (Fig. 12B) and (GE → Bch = 1.01) in Arooj (Fig. 12A). This suggest that transcriptional regulation strongly drives metabolic and antioxidant adjustment in both cultivars. Among the biochemical indicators SOD (0.96) and POD (0.99) showed the highest loading in Chakwal, highlighting the critical role of antioxidant defense in mitigating oxidative stress (Fig. 12B). In contrast, Si accumulation in roots (0.96) and leaves (0.94) showed the highest loadings in Arooj, indicating that Si accumulation plays a dominant biochemical role in this genotype (Fig. 12A). Proline accumulation (0.77) was also strongly associated with drought tolerance, functioning as an osmoprotectant that stabilizes proteins and cellular membranes while scavenging ROS. Si further enhances biochemical resilience by strengthening antioxidant systems and reducing oxidative damage during drought stress.

#### Biochemical control of physiological processes

Biochemical processes exert a moderate positive influence on physiological performance (Bch → Phy = 0.78) in Chakwal and (Bch → Phy = 0.45) in Arooj. The physiological latent construct presented by transpiration rate (TR), water use efficiency (WUE), electron transport/assimilation (ETA), net assimilation rate (NAR), and water potential (WP). Among physiological indicators NAR (0.97), TR (0.97), WUE (0.95), WP (0.92) and ETA (0.90) showed the highest loading in Chakwal (Fig. 12B). While TR (0.98), NAR (0.97), WP (0.95) and WUE (0.92) showed the highest loading in Arooj (Fig. 12A). These results suggest that photosynthetic efficiency and carbon assimilation and osmotic potential processes are the dominant physiological drivers of plant performance under drought stress. If supplementation improves stomatal behavior and leaf structure, it likely enhances carbon assimilation while reducing excessive water loss.

The goodness-of-fit statistics indicated that the structural equation models adequately represented the relationships among the studied variables for both cultivars. The SEM for Chakwal showed an acceptable model fit with a Chi-square/df value of 2.761, a Comparative Fit Index (CFI) of 0.93, and a Tucker–Lewis Index (TLI) of 0.986. Similarly, the SEM developed for Arooj demonstrated a strong model fit with a Chi-square/df value of 2.448, CFI of 0.97, and TLI of 1.053. In addition, the Root Mean Square Error of Approximation (RMSEA) values were low for both Chakwal (0.041) and Arooj (0.036), indicating minimal approximation error (Table S3). The Standardized Root Mean Square Residual (SRMR) values were also within acceptable limits, with values of 0.069 for Chakwal and 0.053 for Arooj. These fit indices confirm that the proposed SEM models for both cultivars provided a reliable representation of the relationships among molecular, biochemical, physiological, and growth-related variables under drought stress and Si treatments.

#### Physiological regulation of morphological traits and yield

Morphological and yield traits are also significantly influenced by physiological processes. NAR (0.97) and ETA (0.89) exhibited strong relationship with plant morphology and yield. This indicates that This indicates that carbon assimilation capacity and photosynthetic electron transport are the primary determinants of yield formation under drought stress.

#### Integrated pathways affecting yield

The SEM clearly illustrates a multilevel regulatory pathway as “Gene Expression → Biochemical Defense → Physiological Performance → Yield Formation”. The strong indirect pathway from gene expression to yield via biochemical and physiological processes suggests that drought tolerance in the Chakwal genotype is largely mediated through coordinated molecular and metabolic regulation. Si supplementation appears to reinforce this cascade by improving osmotic adjustment (proline), antioxidant capacity (POD), and photosynthetic performance.

Overall, the SEM demonstrate that drought tolerance in both cultivars is driven by aquaporin-mediated gene regulation that triggers biochemical defense mechanisms, ultimately stabilizing physiological processes essential for carbon assimilation and yield formation. Si plays a crucial role in mitigating stress by enhancing both biochemical and physiological resilience, particularly through improved proline accumulation, antioxidant activity, and photosynthetic efficiency. Consequently, the integration of molecular, biochemical, and physiological traits forms a coordinated network that enables the cultivars to sustain productivity under water-deficit conditions.

## Discussion

Drought stress is one of the most destructive abiotic factors that limit plant growth and productivity worldwide, especially in arid and semi-arid regions like Balochistan, Pakistan, where soil water availability governs physiological functioning and crop yield^35^. This study was designed to evaluate the effects of different levels of drought stress and Si supplementation on the physiological, morphological, and molecular responses of two mash bean cultivars. Drought stress significantly restricted the growth, biomass, reproductive traits, photosynthetic performance, water relations, and mineral nutrition of both cultivars (Fig. 1–6). However, Si supplementation effectively alleviated these detrimental effects through coordinated molecular, biochemical, physiological, and morphological mechanisms, highlighting its beneficial role in enhancing plant tolerance to drought stress, as reported in numerous plant species^13,36,37^.

The significant reductions in plant height, leaf expansion, and vegetative biomass under drought (Figs. 1A–H and 2C–F) stem from decreased turgor pressure, stomatal closure, and impaired carbon assimilation^38–42^. Elevated abscisic acid (ABA) levels likely altered resource allocation, prioritizing survival over growth^43–45^. Notably, Arooj displayed superior drought tolerance over Chakwal by maintaining greater leaf area and biomass under water deficits, indicating inherent genotypic differences. Si supplementation significantly restored vegetative growth, likely because Si deposition in cell walls strengthens structural rigidity, prevents cell collapse, and maintained cell turgor by improving water uptake^46,47^. This structural reinforcement enabled sustained leaf expansion and biomass accumulation under stress, aligning with previous findings^42,48,49^.

Accelerated phenological development under 25% FC indicates a drought escape strategy where plants accelerate flowering and maturity to prioritize reproduction (Fig. 3A–F)^50,51^. Despite this strategy. Severe drought ultimately compromised pod number, pods clusters, and 100‑seed weight due to source limitations and carbohydrate shortages caused by reduced photosynthesis^52^. Si supplementation successfully mitigated these yield losses by extending flowering and maturity durations and improving reproductive trait. This yield stability under stress is attributed to enhanced photosynthetic capacity and stable assimilate supply supported by Si, consistent with its documented role in improving carbon metabolism under water-limited conditions^53,54^. Drought stress significantly altered plant water relations, reducing water potential and osmotic adjustment (Fig. 4). These disruptions represent a primary physiological constraint that limits stomatal opening, hydraulic conductivity, and leaf gas exchange^55,56^. Conversely, Si supplementation significantly mitigated these effects, improving pressure potential and internal water status compared with non-supplemented plants under drought. This Improvement is attributed to Si deposition within plant tissues, which strengthens cell walls to enhance water retention, reduces transpirational water loss, and increases root hydraulic conductivity^57,58^. This enhanced hydraulic regulation stabilized internal water transport when soil moisture becomes limiting.

This improved hydraulic functioning directly supported photosynthetic performance. Progressive drought significantly reduced net assimilation rate, transpiration rate, and stomatal conductance, reflecting stomatal limitations on carbon assimilation (Fig. 4D–H). The observed increase in intercellular Ci under severe stress indicates non-stomatal limitations, likely driven by impaired Rubisco activity^59^. This reduction in gas exchange restricts ATP and NADPH regeneration, causing feedback inhibition of the Calvin cycle, elevating electron pressure, and oxidative damage to the photosystems^60^. While elevated WUE indicates a conservative water-use strategy, it ultimately reflected suppressed carbon fixation^61^, which was compounded by a decline in photosynthetic pigments (Chl *a*, Chl *b*, carotenoids).

Si supplementation significantly improved photosynthetic performance under drought, maintaining higher net assimilation rates and stomatal conductance (Fig. 4D-H). This preservation of stomatal function suggests that Si-mediated improvements in plant water status and hydraulic conductivity sustained CO₂ diffusion despite limited soil moisture^62,63^. This is further supported by the coordinated responses observed among aquaporin expression, water relations, and gas exchange, demonstrating that enhanced water transport stabilizes leaf hydration and stomatal regulation under stress^64,65^. Between the two cultivars, Arooj showed superior photosynthetic resilience over Chakwal, driven by its higher gas exchange capacity and superior pigment retention under stress.

The transcriptional upregulation of *DREB2A*, *PIP2-1*, and *TIP4-1* under drought reflects the activation of stress-responsive networks (Fig. 8B-D). *DREB2A* act as a key transcription factor regulating dehydration-responsive genes to promote osmolytes and protective protein synthesis^66,67^. Crucially, drought stress induced the upregulation of aquaporin genes *PIP2-1* and *TIP4-1*, an adaptive response to regulate membrane water permeability and enhance radial water transport and vaculor water redistribution under water deficits^68,69^. Si supplementation further amplified the expression of these aquaporin genes, suggesting that Si may modulates water transport pathways at the molecular level. This coordinated regulation of aquaporins directly underpins the improved plant water relations hydraulic conductivity observed in this study, enabling maintained cellular hydration and sustained physiological functioning under drought^68^. Between the two cultivars, Arooj exhibited consistently higher gene expression levels than Chakwal. This robust molecular response correlates with its superior physiological and morphological performance, confirming that genotype‑dependent regulation of drought‑responsive genes dictates overall stress tolerance in mash bean.

RWC, osmotic potential, water potential, and leaf turgor declined with increasing drought intensity, particularly at 25% FC, reflecting cellular stress (Fig. 4A–C). Maintaining turgor is critical for continued cell expansion and photosynthetic activity^41^. Si supplementation improved RWC, osmotic adjustment, and turgor potential, by facilitating solute accumulation (e.g., proline, sugars) and stabilizing cellular membranes. Enhanced turgor under Si treatment supports sustained physiological performance and biomass accumulation despite reduced soil moisture^19^. In addition to hydraulic regulation, drought stress also triggered significant increases in antioxidant enzyme activities, including SOD, CAT, MDA and APX, as well as increased accumulation of phenolic compounds (Fig. 5B–E). These biochemical responses represent protective mechanisms that help mitigate oxidative stress generated during drought conditions. Water deficit often leads to disruption of electron transport processes and increased formation of ROS, which can damage cellular structures and metabolic pathways^70,71^. The enhanced antioxidant activity observed in Si-treated plants suggests that silicon contributes to maintaining cellular homeostasis by strengthening the antioxidant defense system. Increased antioxidant capacity may help protect photosynthetic machinery and cellular membranes from oxidative damage during periods of limited water availability. Although antioxidant responses represent a secondary protective mechanism, they likely complement hydraulic and physiological adjustments to maintain metabolic stability under drought stress. Accumulation of phenolics and other non‑enzymatic antioxidants under drought suggests activation of secondary metabolic pathways such as the phenylpropanoid pathway, which contribute to ROS scavenging and cell wall reinforcement^72^. The greater antioxidant responses in Arooj imply a more constitutive or inducible defense network, which contributes to its superior stress tolerance. Si further stimulated antioxidant activities and osmolyte accumulation (proline, TSP). This enhancement likely arises from Si mediated modulation of stress signaling pathways, enabling more efficient ROS detoxification and osmotic adjustment. Elevated proline not only contributes to osmotic balance but also acts as a ROS scavenger, stabilizing proteins and membranes under dehydration^73,74^.

Drought stress disrupted the uptake and translocation of essential minerals (K, Ca, Mg, P, Fe, Zn, Cu), likely due to reduced transpiration and impaired root absorption (Fig. B7–H). Reduced mineral nutrition can compound physiological stress by limiting enzyme activities and osmotic adjustment. Si supplementation significantly increased leaf nutrient concentrations under stress, supporting the notion that Si enhances root water flux and nutrient uptake^75,76^. Minerals such as potassium play key roles in osmotic regulation and stomatal movement, while Ca stabilizes membrane structure and cell signaling under stress^77,78^. Therefore, improved mineral nutrition in Si treated plants likely underpinned enhanced water relations and growth.

PCA and hierarchical clustering revealed distinct separations between treatment groups, where Si supplemented plants under moderate stress clustered closely with controls, characterizing the restorative capacity of Si on biomass, leaf area, and chlorophyll content (Fig. 9B–D). Conversely, severe drought treatments associated strongly with elevated WUE, intercellular CO_2_, and ETR/A, reflecting adaptive physiological conservation. Correlation matrices further confirmed strong synergistic relationships among Si supplementation, antioxidant defense, phenolic, and nutrient accumulation, alongside inverse correlations between water/osmotic potentials and drought-responsive gene expression (Figs. 10,11)^79^. SEM analysis validated a hierarchical cascade: Gene Expression → Biochemical Defense → Physiological Performance → Morphology and Yield (Fig. 12A, B). With this network, *TIP4-1* and *PIP2-1* expression exerted a strong influence on hydraulic regulation and gene-mediated biochemical adjustments. these biochemical responses (antioxidants, proline and Si accumulation) subsequently governed downstream physiological traits, ultimately dictating vegetative growth and reproductive yield. Si supplementation significantly reinforced these integrated pathways, stabilizing osmotic potential, and sustaining photosynthesis to preserve final yield^13,80–82^. This multilevel integration confirmed that the drought tolerance of Arooj over Chakwal is driven by more efficient aquaporin genes regulation, higher antioxidant and phenolic capacities, better osmotic adjustment, and improved mineral nutrition. SEM and PCA models conclusively demonstrate that Arooj integrates these molecular, metabolic, and physiological pathways more effectively, thereby sustaining cellular turgor, vegetative growth, and reproductive productivity under severe water deficits.

## Conclusion

The present study evaluated the responses of two *Vigna mungo* cultivars to drought stress and Si supplementation using integrated morphological, physiological, biochemical, and molecular analyses. Drought stress significantly reduced plant growth, biomass and reproductive performance, photosynthetic gas exchange, and plant water relations, while enhancing activities of antioxidant enzymes, phenolic compounds, and expression of drought-responsive genes (*DREB2A*, *PIP2-1* and *TIP4-1*), indicating activation of stress defense mechanisms. Si supplementation improved growth attributes, biomass production, reproductive traits, and photosynthetic gas exchange characteristics under drought stress. Si treated plants also exhibited improved water status, enhanced antioxidant enzyme activities, increased accumulation of phenolic compounds, and higher expression of drought-responsive and aquaporin genes. Multivariate analyses further demonstrated clear differentiation among treatments, with Si-supplemented plants clustering closer to well-watered conditions. Structural equation modeling identified significant relationships linking gene expression, biochemical responses, physiological processes, and plant growth traits under drought conditions. The data demonstrate that Si supplementation significantly improves drought tolerance in mash bean by enhancing plant growth performance, maintaining physiological stability, strengthening antioxidant defense systems, and regulating the expression of key drought-responsive genes. Among the tested cultivars, Arooj consistently showed greater resilience to drought stress compared with Chakwal across most measured traits.

## Supplementary Information

Supplementary Information.

## Data Availability

Data will be made available on formal request to corresponding author.
