# Supplementary material for: Silicon-mediated drought tolerance in Vigna mungo through coordinated regulation of aquaporin genes, plant water relations and photosynthetic processes
Source: Sci Rep. 2026 Jul 9;16:28746. doi: 10.1038/s41598-026-59877-x (PMC13578461; doi:10.1038/s41598-026-59877-x)
Supplement: Supplementary file 1 [file 41598_2026_59877_MOESM1_ESM.pdf]

Supplementary Material

Table 1. List of primers used in the current study

Gene name	Forward (5′-3′)	Reverse (5′-3′)
**DREB2A**	CTGCTCTTGCTTATGATGAA	ATGTAGTGGTAGTAGTAGTAGTG
**PIP2-1**	TGACCCAGTGGAGCTTCTA	GGCTCTTGTAGCCGATCAC
**TIP1-3**	CCGGAATTCATGGACACCAAGCATGCGGA	TCCCCCGGGCTAAAAGTCCCAACCAAGTA

Table 2. Analysis of variance (ANOVA) of different morphological, physiological, biochemical and molecular traits studied in both Arooj and Chakwal.

**Source**	**df**	**PH**	**LL**	**LW**	**LA**	**NOL_P**	**SL**	**RL**	**DF_50**	**DF_75**	**DF_90**	**DM_50**	**DM_75**	**DM_90**
		F(p)	*F(p)*	*F(p)*	*F(p)*	*F(p)*	*F(p)*	*F(p)*	*F(p)*	*F(p)*	*F(p)*	*F(p)*	*F(p)*	*F(p)*
**Genotype**	1	77.8***	107***	58.2***	122.9***	9.33**	50.8***	29.8***	25.1***	142.3***	68.4***	440***	587.9***	1917***
**Treatment**	6	759***	55.5***	99.1***	121.1***	53.5***	25.7***	81***	97.2***	93.2***	63.9***	113.6***	154.8***	280.8***
**Gen × Trt**	6	8.97*	2.58*	4.05***	1.99n.s.	0.90n.s.	3.17*	0.81n.s.	0.04*	0.00***	1.64n.s.	3.28*	3.66**	20.18***
**Residuals (Error)**	28													
	**df**	**PDL**	**NOP/P**	**SW**	**NOCP**	**NOPC**	**RFW**	**RDW**	**SFW**	**SDW**	**Chla**	**Chlb**	**Car**	**SOD**
		F(p)	*F(p)*	*F(p)*	*F(p)*	*F(p)*	*F(p)*	*F(p)*	*F(p)*	*F(p)*	*F(p)*	*F(p)*	*F(p)*	*F(p)*
**Genotype**	1	101.6***	109***	97.3***	41.6***	1.6n.s.	20.8***	31.4***	62.5***	48.3***	35.3***	188.9***	84.7***	17.5***
**Treatment**	6	32.0***	116***	265***	52.0***	15.33***	133***	90***	141***	120***	108***	80.5***	98.2***	136***
**Gen × Trt**	6	1.58n.s.	0.49n.s.	1.47n.s.	0.40n.s.	1.13n.s.	0.83n.s.	0.20n.s.	0.43n.s.	3.05*	0.26n.s.	6.74***	0.03*	0.48n.s.
**Residuals (Error)**	28													
	**df**	**POD**	**CAT**	**APX**	**TSP**	**Proline**	**RWC**	**OP**	**WP**	**PP**	**NAR**	**Ci**	**TR**	**SC**
		F(p)	*F(p)*	*F(p)*	*F(p)*	*F(p)*	*F(p)*	*F(p)*	*F(p)*	*F(p)*	*F(p)*	*F(p)*	*F(p)*	*F(p)*
**Genotype**	1	13.9***	13.8***	81.5***	46.0***	42.2***	60.4***	41.3***	0.52n.s.	15.5***	3.91n.s.	0.00n.s.	1.01n.s.	15.1***
**Treatment**	6	482***	456***	134.1***	161.4***	81.7***	113***	108***	50.5***	28.7***	124.8***	16.1***	95.6***	126.3***
**Gen × Trt**	6	0.45n.s.	0.08n.s.	2.18n.s.	0.64n.s.	0.33n.s.	1.02n.s.	0.89n.s.	3.2*	1.60n.s.	0.32n.s.	0.32n.s.	0.47n.s.	0.26n.s.
**Residuals (Error)**	28													
	**df**	**ETA**	**WUA**	**K**	**Ca**	**Mg**	**Ph**	**Su**	**Bo**	**Cu**	**Fe**	**Mn**	**Zn**	**GA**
		F(p)	*F(p)*	*F(p)*	*F(p)*	*F(p)*	*F(p)*	*F(p)*	*F(p)*	*F(p)*	*F(p)*	*F(p)*	*F(p)*	*F(p)*
**Genotype**	1	13.4***	4.51*	3.91*	12.6***	95.6***	79.8***	41.4***	16.31***	2.34n.s.	7.90***	6.18**	12.9**	56.9***
**Treatment**	6	22.6***	43.5***	13.6***	41.4***	45.9***	190.5***	29.5***	55.0***	14.7***	54.8***	32.5***	66.5***	129.5***
**Gen × Trt**	6	1.04n.s.	0.61n.s.	0.28n.s.	0.24n.s.	2.12***	2.4**	1.17n.s.	1.20n.s.	0.48n.s.	2.97**	1.95**	2.34**	2.53*
**Residuals (Error)**	28													
	**df**	**CA**	**FA**	**Qu**	**VA**	**DR**	**PIP**	**TIP**	**Si_L**	**Si_R**	**MDA**			
		F(p)	*F(p)*	*F(p)*	*F(p)*	*F(p)*	*F(p)*	*F(p)*	*F(p)*	*F(p)*	*F(p)*			
**Genotype**	1	213***	68.3***	42.4***	37.8***	61.5***	102.9***	26.6***	9.01**	8.81**	33.7***			
**Treatment**	6	95.2***	82.2***	81.2***	196.7***	21.69***	57.73***	12.18***	81.53***	122***	47.1***			
**Gen × Trt**	6	8.06*	9.96**	3.2**	2.55*	3.16**	7.88***	3.74**	2.84*	2.15*	1.97*			

**Residuals (Error)**	28													

Table 3. Metric Model fit values to support SEM of Arooj and Chakwal.

Metric	Chakwal	Arooj
Chi-square/df	2.761	2.448
CFI (Comparative Fit Index)	0.93	0.97
TLI (Tucker-Lewis Index)	0.986	1.053
RMSEA (Root Mean Square Error of Approximation)	0.041	0.036
SRMR (Standardized Root Mean Square Residual)	0.069	0.053

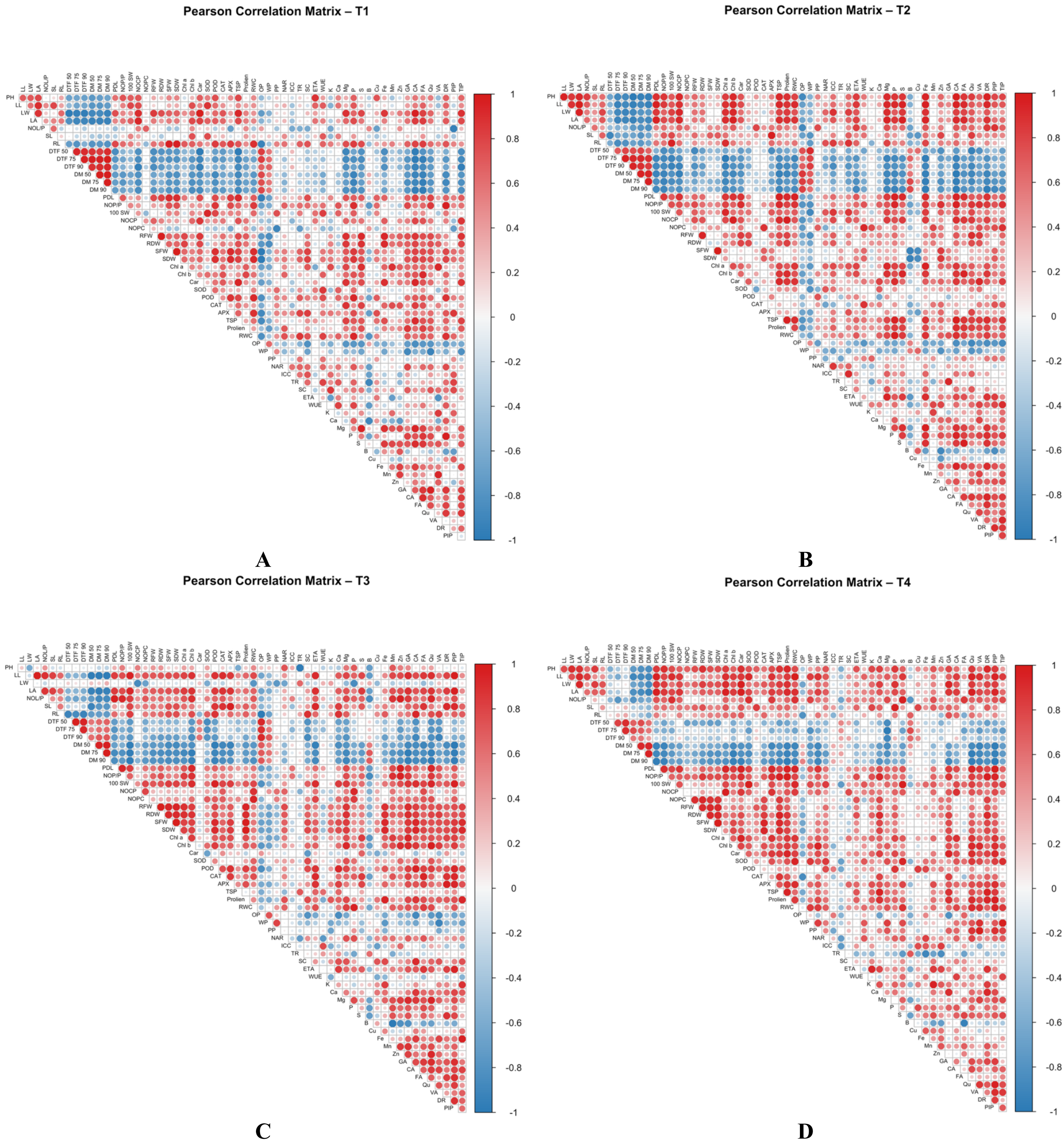

Figure 1. Pearson correlation analysis displayed the relationship among all the studied traits under different levels of drought stress and Si supplementation. For each treatment a separate correlation was performed to better understand the treatment effect and behavior of traits.

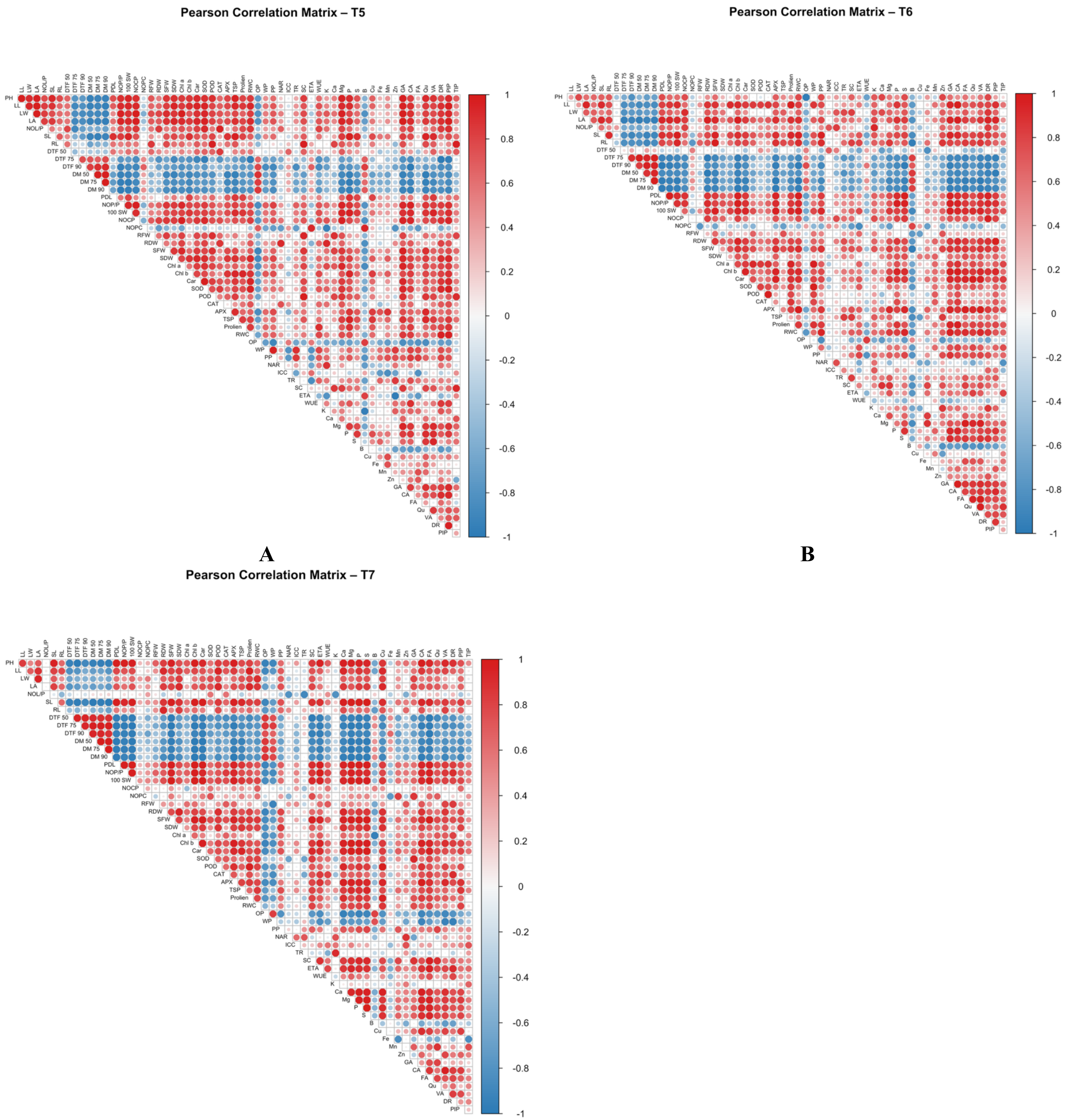

Figure 2. Pearson correlation analysis displayed the relationship among all the studied traits under different levels of drought stress and Si supplementation. For each treatment a separate correlation was performed to better understand the treatment effect and behavior of traits.
